# Synthesis and Fluorescence Properties of a New Heterotrinuclear Co(II)-Ce(III)Complex Constructed from a bis(salamo)-Type Tetraoxime Ligand

**DOI:** 10.3390/molecules23040804

**Published:** 2018-03-31

**Authors:** Lu-Mei Pu, Qing Zhao, Ling-Zhi Liu, Han Zhang, Hai-Tao Long, Wen-Kui Dong

**Affiliations:** 1 College of Science, Gansu Agricultural University, Lanzhou 730070, China; dapanji@163.com; 2 School of Chemical and Biological Engineering, Lanzhou Jiaotong University, Lanzhou 730070, China; zq18215194507@163.com (Q.Z.); llz1009663202@126.com (L.-Z.L.); 13572510846@163.com (H.Z.)

**Keywords:** bi(salamo)-type ligand, heterotrinuclear complex, synthesis, crystal structure, fluorescence property

## Abstract

[Co_2_(L)Ce(OAc)_3_(CH_3_CH_2_OH)]·1.5CH_3_OH∙0.5CH_2_Cl_2_, a heterotrinuclear Co(II)-Ce(III) bis(salamo)-type complex with a symmetric bi(salamo)-type ligand H_4_L and an acyclic naphthalenediol moiety, was designed, synthesized and characterized by elemental analyses, FT-IR, UV-Vis and fluorescence spectroscopy and X-ray crystallography. The X-ray crystallographic investigation revealed the heterotrinuclear complex consisted of two Co(II) atoms, one Ce(III) atom, one (L)^4‒^ unit, three *μ*_2_-acetate ions, one coordinated ethanol molecule, one and half crystallization methanol molecule and half crystallization dichloromethane molecule. Two Co(II) atoms located in the N_2_O_2_ coordination spheres, are both hexacoordinated, with slightly distorted octahedral geometries. The Ce(III) atom is nine-coordinated and located in the O_6_ cavity possesses a single square antiprismatic geometry. In addition, supramolecular interactions exist in the Co(II)-Ce(III) complex. Two infinite 2D supramolecular structures are built via intermolecular O–H···O, C–H···O and C–H···*π* interactions, respectively.

## 1. Introduction

Salen (*N*,*N*-disalicylideneethylenediamine) is a versatile important compound that has been widely used in coordination chemistry and organometallic chemistry [1,2,3,4,5,6,7,8]. On the one hand, salen-type compounds are tetradentate ligands with a N_2_O_2_ coordination environment that can usually coordinate with transition metal ions to afford diverse metal complexes. On the other hand, a number of salen-type metal complexes have already been synthesized to study their structures and applied in various fields for their magnetic properties [9,10,11,12,13,14], catalytic action [15,16], electrochemistry [17,18], in biological systems [19,20,21,22,23,24,25,26,27,28], supramolecular architectures [29,30,31,32,33,34,35,36,37], their luminescent properties [38,39,40,41,42,43,44], and in optical sensors [45] and nonlinear optical materials [46].

More recently, salamo-type ligands [47,48,49,50,51,52,53,54,55,56] using an *O*-alkyloxime (–CH=N–O–(CH_2_)*_n_*–O–N=CH–) have been reported. In our previous studies on salamo-type metal complexes, we exchanged salicylaldehyde for its derivatives to obtain some new salamo-type complexes with different structures. Our research group is focused on the synthesis and study of 3d–4f heterometallic salamo-type complexes [14,16,49].

**Figure 1:**
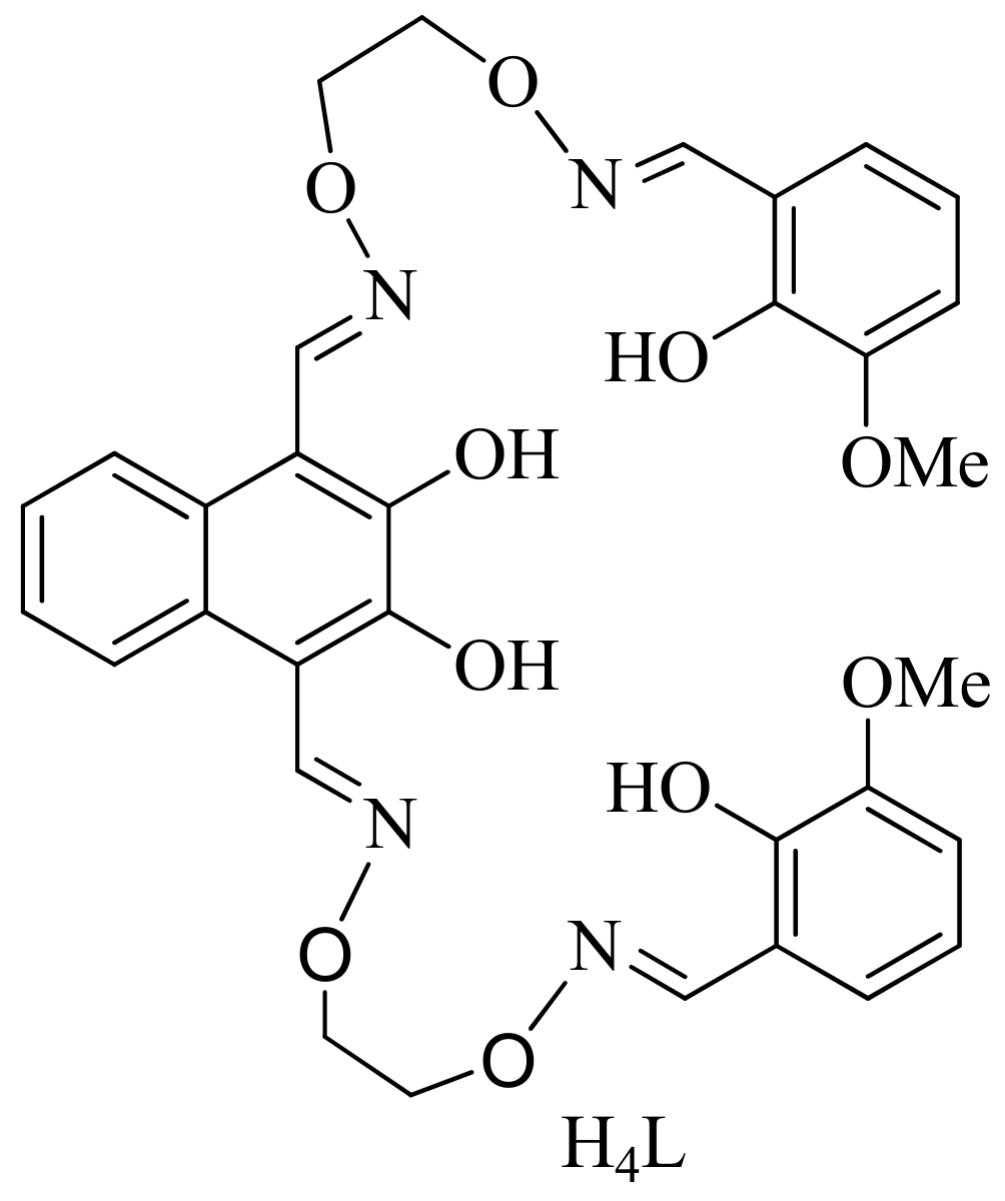
Structural representation of the ligand H_4_L.

Based on these points of view, we have now designed and synthesized a symmetric bi(salamo)-type ligand H_4_L and its corresponding heterotrinuclear Co(II)-Ce(III) 3d–4f complex [Co_2_(L)Ce(OAc)_3_(CH_3_CH_2_OH)]∙1.5CH_3_OH∙0.5CH_2_Cl_2_. The structure of H_4_L is depicted in Figure 1. Furthermore, the structure and fluorescence properties of the Co(II)-Ce(III) complex were studied.

## 2. Results and Discussion

### 2.1. IR Spectra 

**Figure 2:**
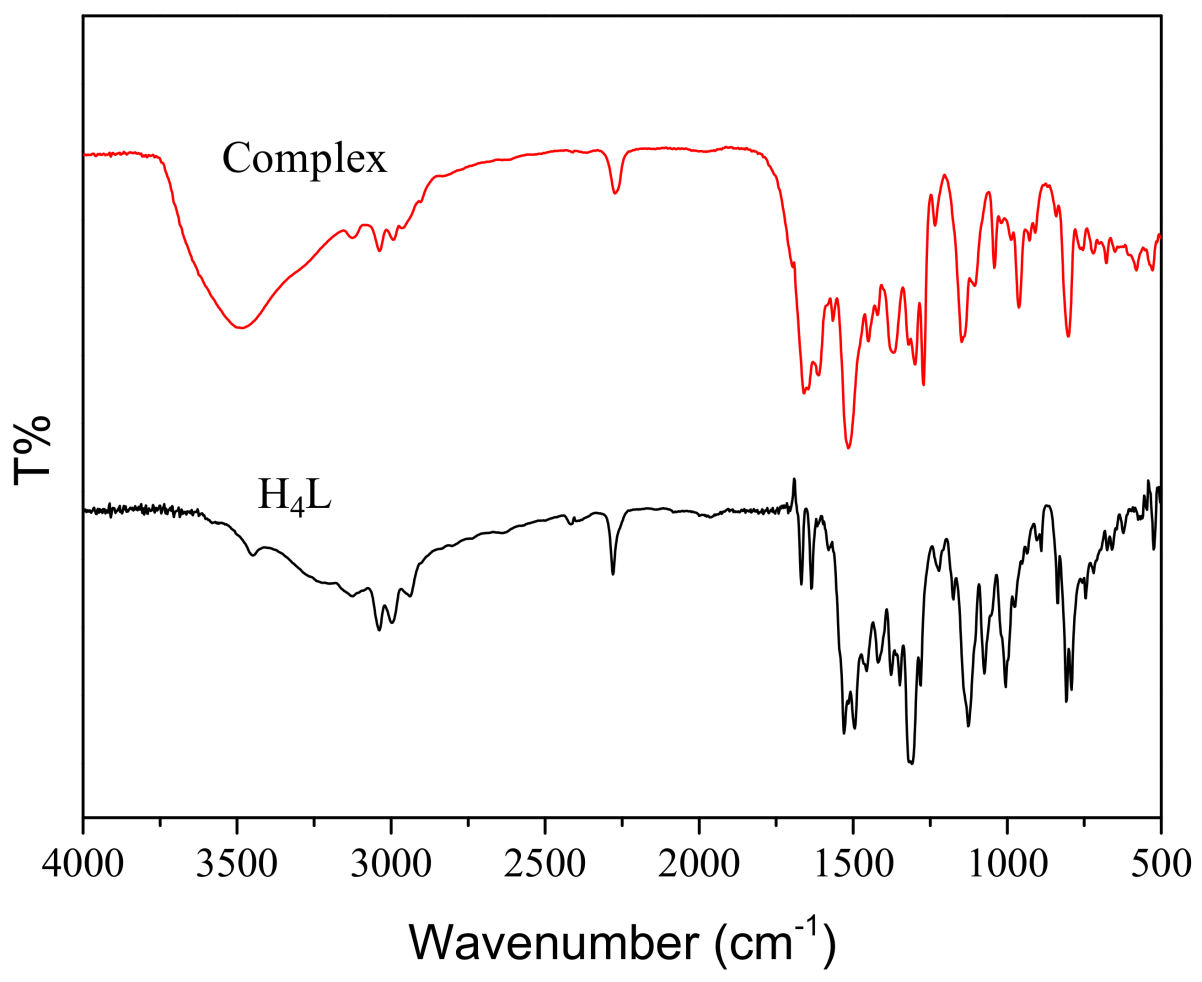
IR spectra of the ligand H_4_L and its complex.

The IR spectra of H_4_L and its corresponding Co(II)-Ce(III) complex exhibited various bands in the 4000–400 cm^−1^ region. The FT-IR spectrum and data of the ligand H_4_L and its corresponding Co(II)-Ce(III) complex are given in Figure 2 and Table 1. 

A C=N stretching vibration band was observed at 1612 cm^−1^ in the IR spectrum of H_4_L. Upon complex formation, the strong characteristic C=N vibration band of the Co(II)-Ce(III) complex appeared at 1623 cm^−1^, which is slightly red-shifted in comparison to the free ligand H_4_L and is attributed to coordination of the nitrogen atoms of the C=N group and the metal(II) atoms [57]. The Ar–O stretching vibration band is detected in a range of 1220–1260 cm^−1^. In the free ligand H_4_L the characteristic Ar–O group absorption appeared at 1255 cm^−1^, a ca. 16 cm^−1^ shift to a lower frequency showing that the M–O bonds are formed between the metal atoms and the oxygen atoms from methoxy and phenolic groups of the free ligand H_4_L [58]. The above facts are consistent with the results determined by X-ray diffraction. 

### 2.2. UV-Vis Absorption Spectra 

In many studies, UV-Vis absorption spectra have been utilized to study lanthanide complexes. In this study, the UV-Vis spectra of the free ligand H_4_L in CHCl_3_:CH_3_OH (1:1) (*c* = 2.5 × 10^−5^ mol L^−1^) with its corresponding Co(II)-Ce(III) complex in CH_3_OH:H_2_O (10:1) (*c* = 1 × 10^−3^ mol L^−1^) were collected in the range of 250–550 nm. In the absorption spectrum of the free ligand H_4_L, there are four consecutive absorption peaks at ca. 269, 342, 360 and 375 nm. The absorption peak at 269 nm can be assigned to the *π*–π* transition of the benzene rings. The other three absorption peaks can be attributed to the *π*–π* transition of the oxime groups [57]. 

**Figure 3:**
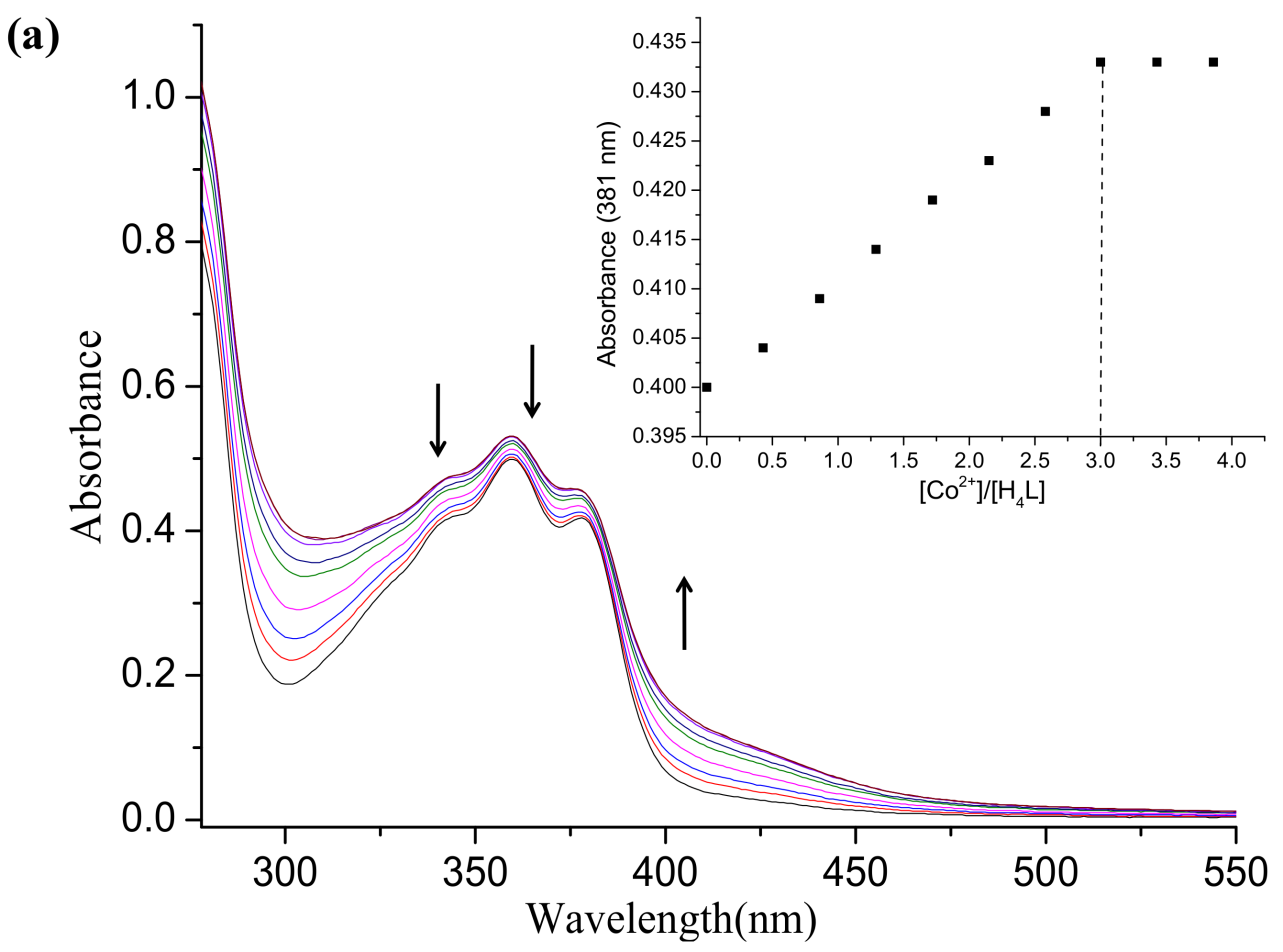

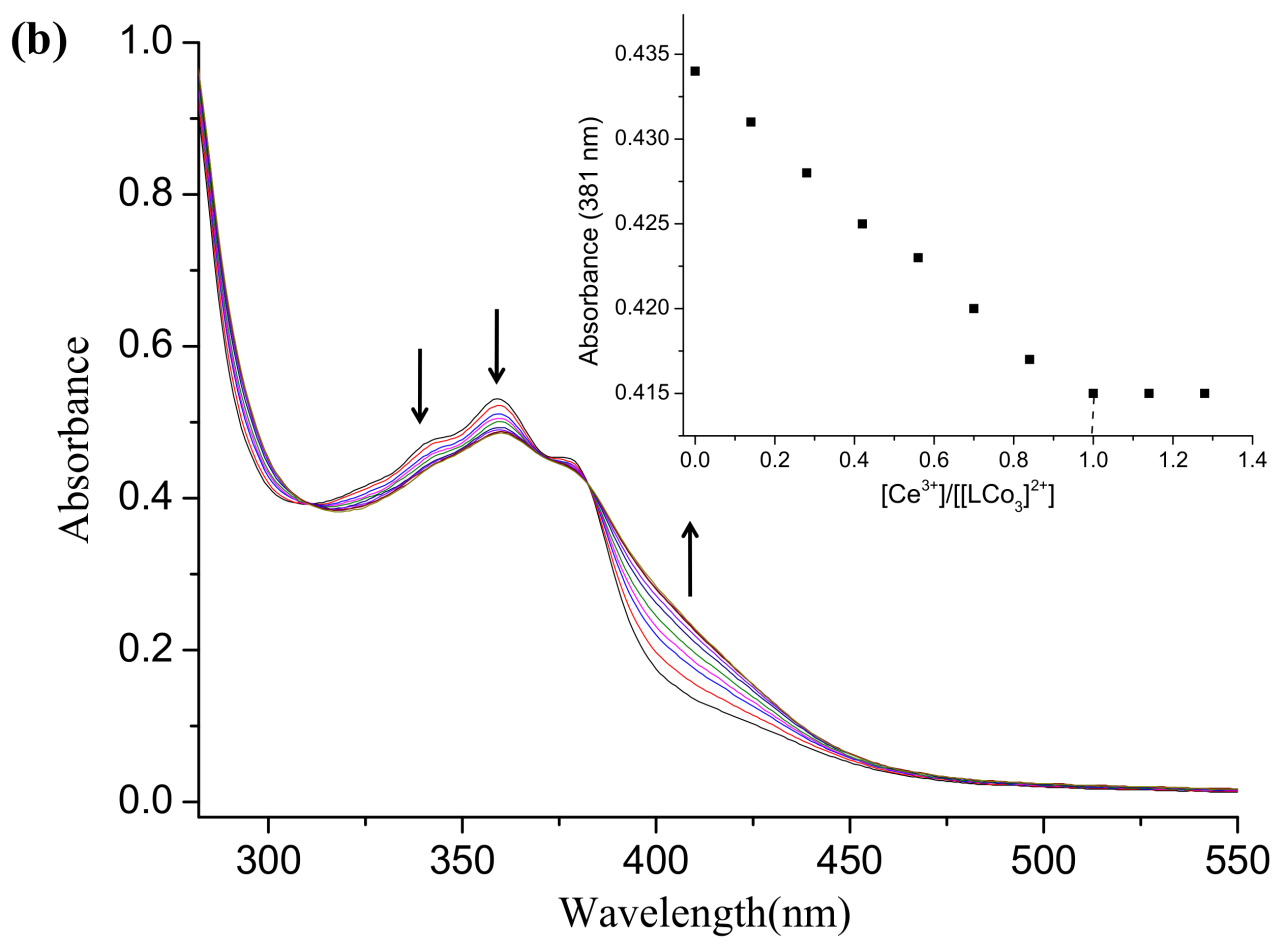
(**a**) UV-Vis spectral changes of the H_4_L (2.5 × 10^−5^ M) on addition of Co(II) (1.0 × 10^−3^ M) ions; (**b**) UV-Vis spectral changes of the [LCo_3_]^2+^ on addition of Ce(III) (1.0 × 10^−3^ M) ions.

In the UV-Vis titration experiment, it can be clearly seen that the gradual addition of Co(OAc)_2_ solution caused absorption peak changes. Compared to the free ligand H_4_L, the absorption peaks are bathochromically shifted [59]. This phenomenon is due to the coordination of H_4_L with the Co(II) ions. Upon addition of Co(II) ions, the absorbance of the solution first increases. When Co(II) ions were added in excess of three equiv, the absorbance of the solution no longer changed. The spectroscopic titration clearly showed the formation of a 1:3 Co(II) complex (Figure 3a).

The color of the solution changed unconspicuously when Ce(III) ions were added. Then, upon addition of 1 equiv of Ce(II) ions, the absorbance changed and showed three isoabsorptive points at about 313, 365 and 380 nm. The spectroscopic titration clearly exhibited that the ratio of the replacement reaction stoichiometry is 1:1 and is shown in Figure 3b.

The importance of the coordination of OAc^−^ was confirmed by the following experiment. A 1:3:1 mixture of H_4_L, Co(NO_3_)_2_ and Ce(NO_3_)_3_ displayed an absorption spectrum identical to that of H_4_L, indicating no complexation. Spectrophotometric titration of the mixture with KOAc showed that nine equiv of KOAc were required to convert the mixture to [LCo_2_Eu(OAc)_3_]. The nine equiv of OAc-consists of six for deprotonation and three for coordination to the trinuclear core.

### 2.3. Crystal Structure Description

**Figure 4:**
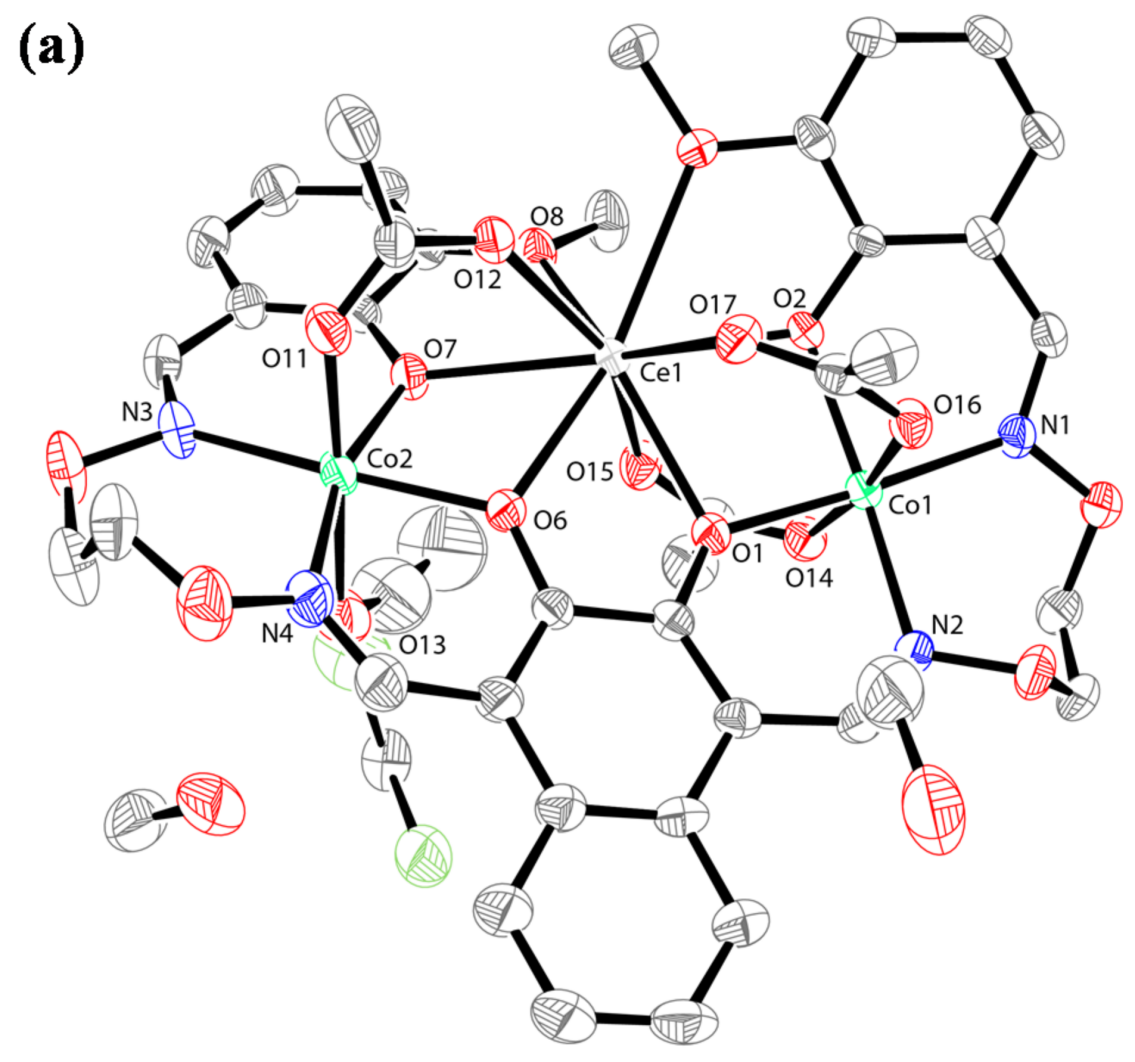

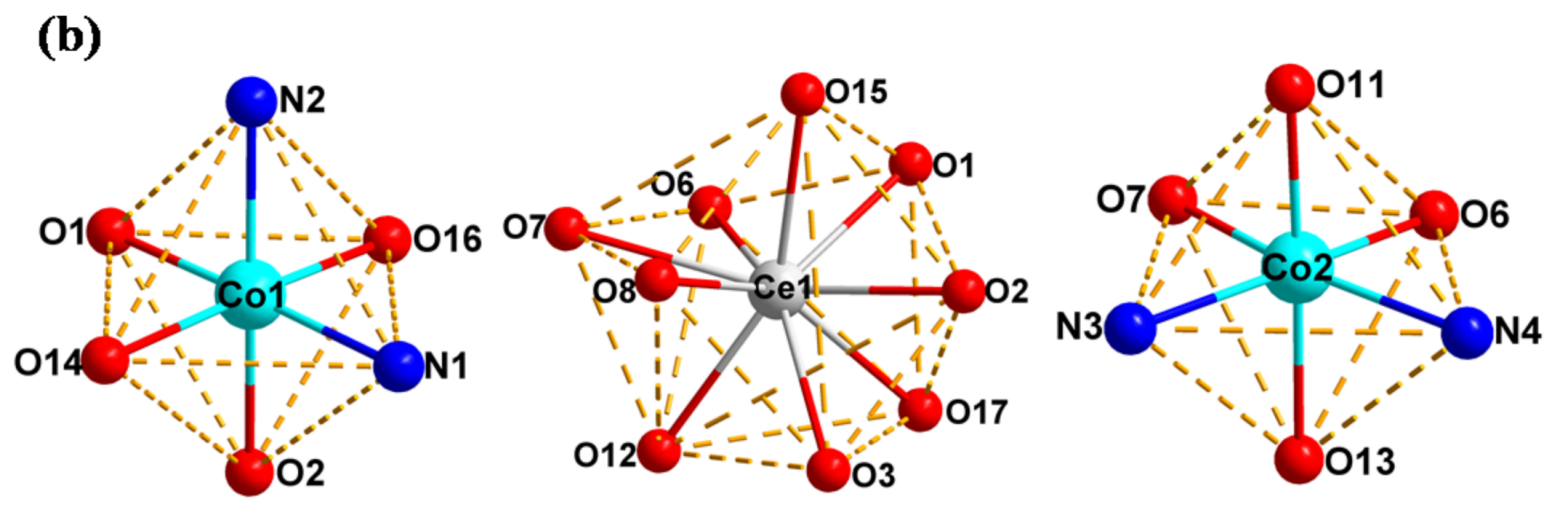
(**a**) View of the molecular structure of the Co(II)-Ce(III) complex; (**b**) Coordination polyhedrons for Co(II) and Ce(III) atoms.

The crystal structure of the Co(II)-Ce(III) complex was determined by X-ray crystallography and is shown in Figure 4. 

X-ray crystallographic analysis revealed that the Co(II)-Ce(III) complex crystallizes in a triclinic system, space group of *P-*1, possessing a symmetric trinuclear structure. The Co(II)-Ce(III) complex consists of two Co(II) atoms, one Ce(III) atom, one (L)^4‒^ unit, three *μ*_2_-acetate ions, one coordinated ethanol molecule, one and half crystallization methanol molecules and half crystallization dichloromethane molecule. As shown in Figure 4, we can see that the coordination ratio of the ligand (L)^4−^ unit to metal atoms (Co(II) and Ce(III)) in the Co(II)-Ce(III) complex is 1:2:1 [60,61]. Meanwhile, the terminal Co(II) atoms (Co1 and Co2) are located in the N_2_O_2_ compartments and they are both hexa-coordinated with slightly distorted octahedral geometries [62,63,64]. Differently, the Co1 atom is bonded to two *μ*_2_-acetate ions (O14 and O16), the nitrogen atoms (N1 and N2) and oxygen atoms (O1 and O2) of the oxime and phenolic groups. The Co2 atom is coordinated the nitrogen atoms (N3 and N4) and oxygen atoms (O6 and O7) of the oxime and phenolic groups, one *μ*_2_-acetate oxygen atom (O11) and one oxygen atom (O13) from the coordinated ethanol molecule. The central O_6_ site (O1, O2, O3, O6, O7 and O8) was occupied by one Ce(III) atom coordinating to three oxygen atoms (O12, O15 and O17) of three *μ*_2_-acetate ions. Hence, the Ce(III) atom is nine-coordinated with a single square antiprismatic geometry. In the crystal structure of the Co(II)-Ce(III) complex, three *μ*_2_-acetate ions bridge Co(II) and Ce(III) atoms in a familiar *μ*_2_-fashion mode. The high coordination number of Ce(III) atom is determined by its longer ionic radius and smaller winding angles. The Ce–O bond distances of the four phenolic oxygen (O1, O2, O6 and O7) atoms from the completely deprotonated (L)^4^^−^ unit are in the range of 2.421(4)–2.523(3) Å and the Ce–O bond distances of the methoxy groups are about Ce1-O3, 2.688(4) and Ce1-O8 2.655(4) Å, which are clearly shorter than later. The crystallographic data and structural refinement paramenters are summarized in Table 2. Selected bond lengths and angles of the Co(II)-Ce(III) complex are listed in Table 3.

### 2.4. Supramolecular Interactions 

Notably, supramolecular interactions exist in the Co(II)-Ce(III) complex. The hydrogen bonds and C–H···*π* stacking interactions are listed in Table 4. 

**Figure 5:**
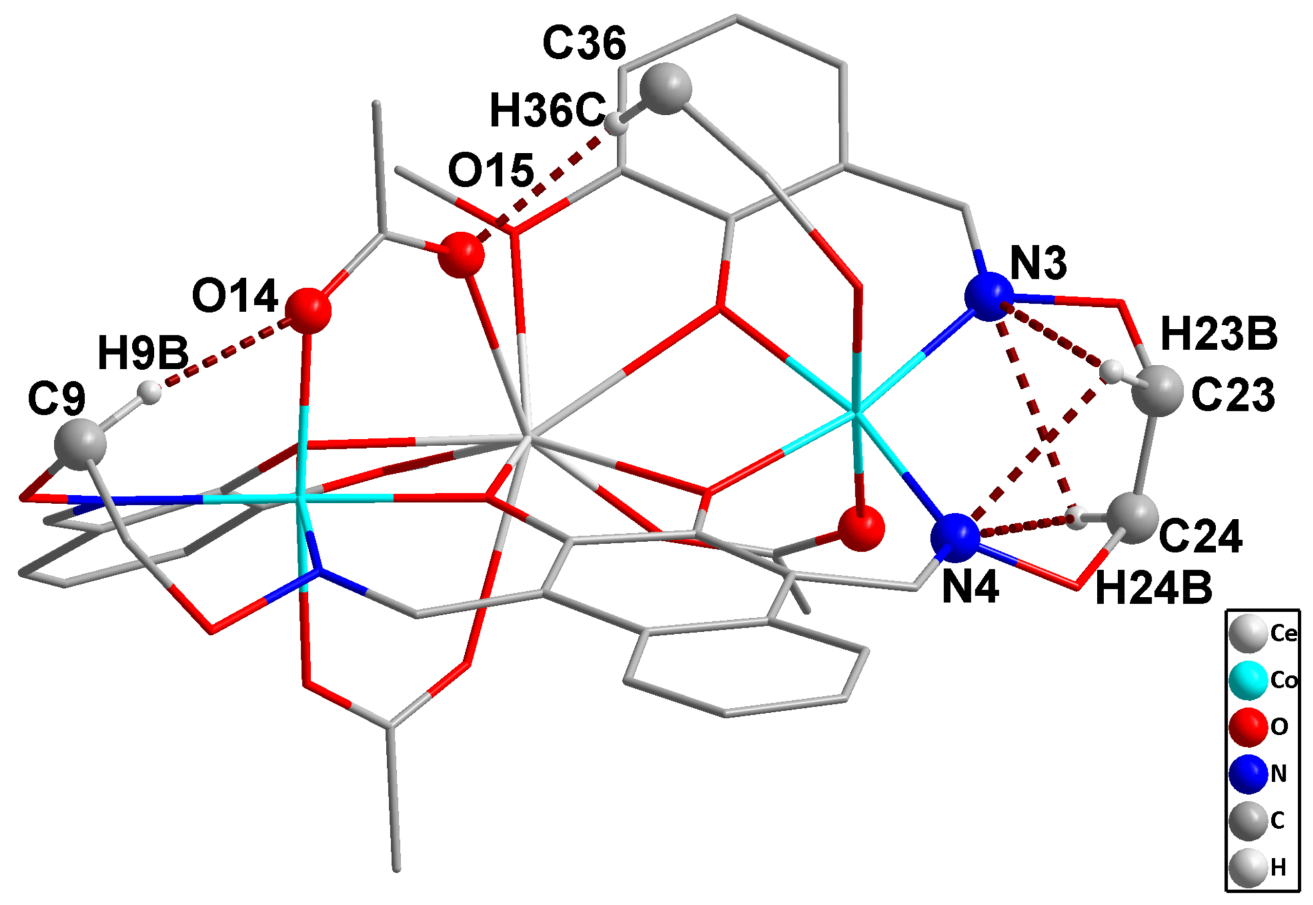
View of the intramolecular hydrogen bonds for the Co(II)-Ce(III) complex.

**Figure 6:**
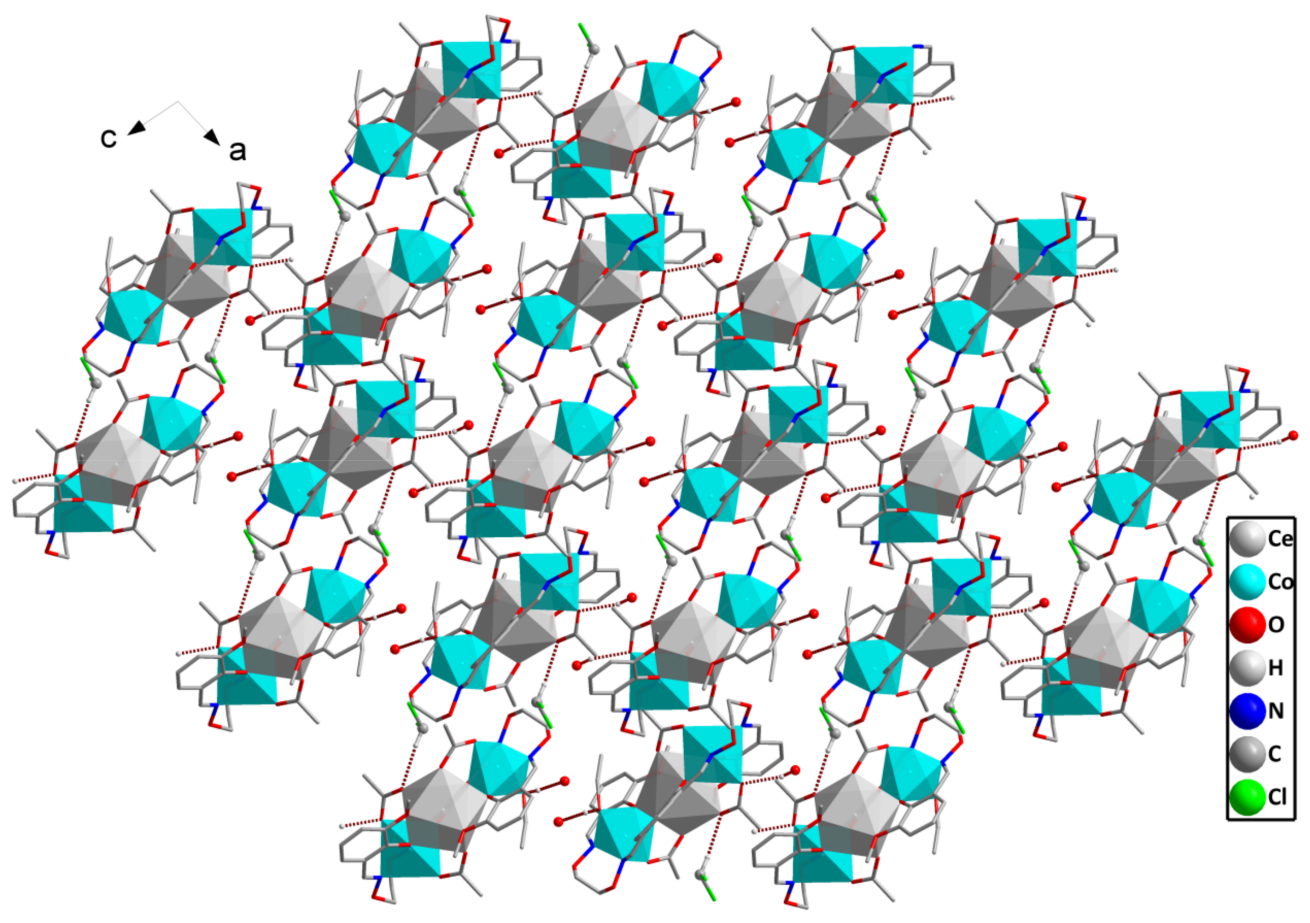
View of 2D supramolecular structure by the intermolecular O–H**···**O and C–H**···**O interactions of the Co(II)-Ce(III) complex.

**Figure 7:**
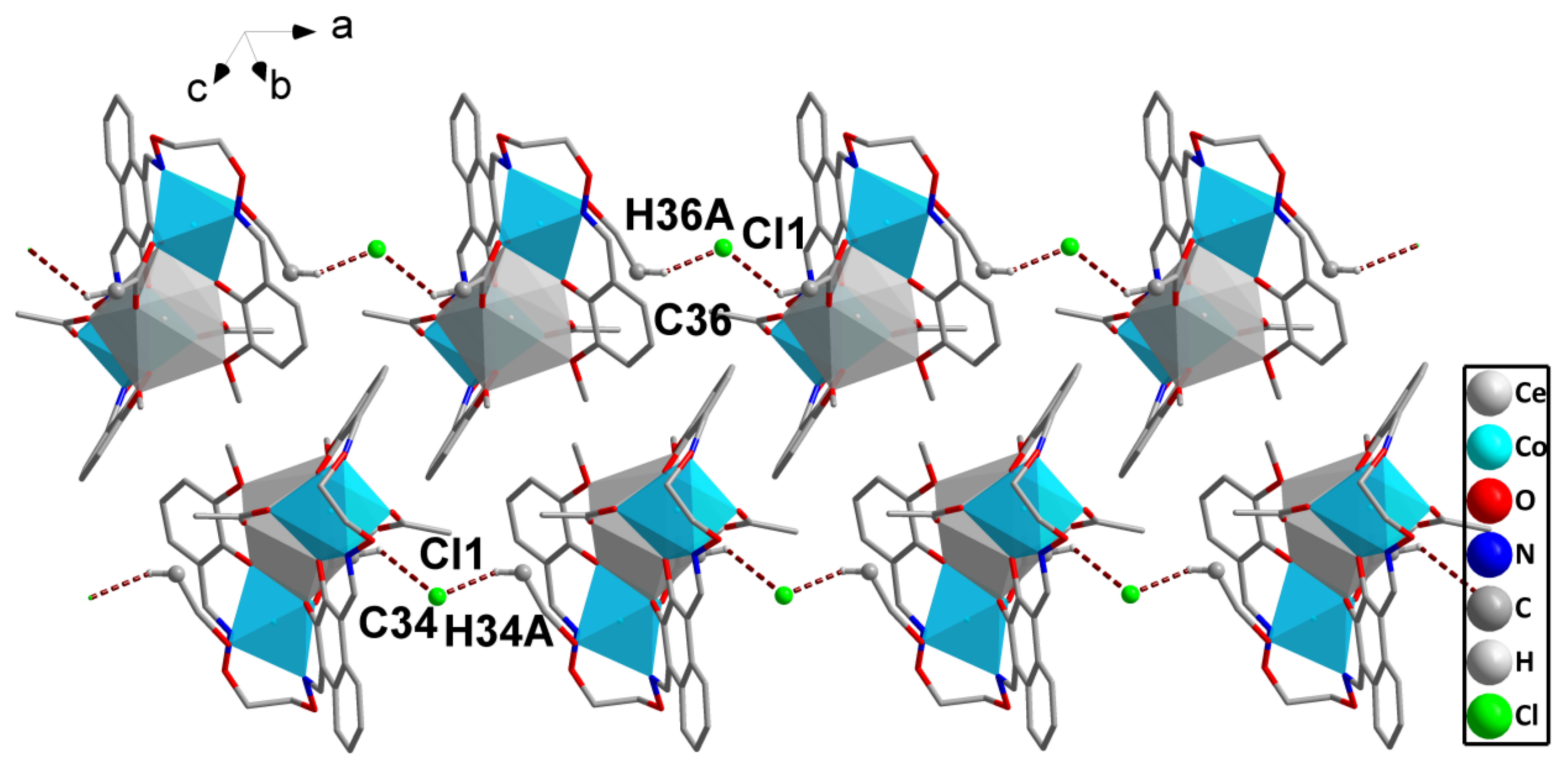
View of 1D supramolecular structure by the intermolecular C–H**···**Cl interactions of the Co(II)-Ce(III) complex.

**Figure 8:**
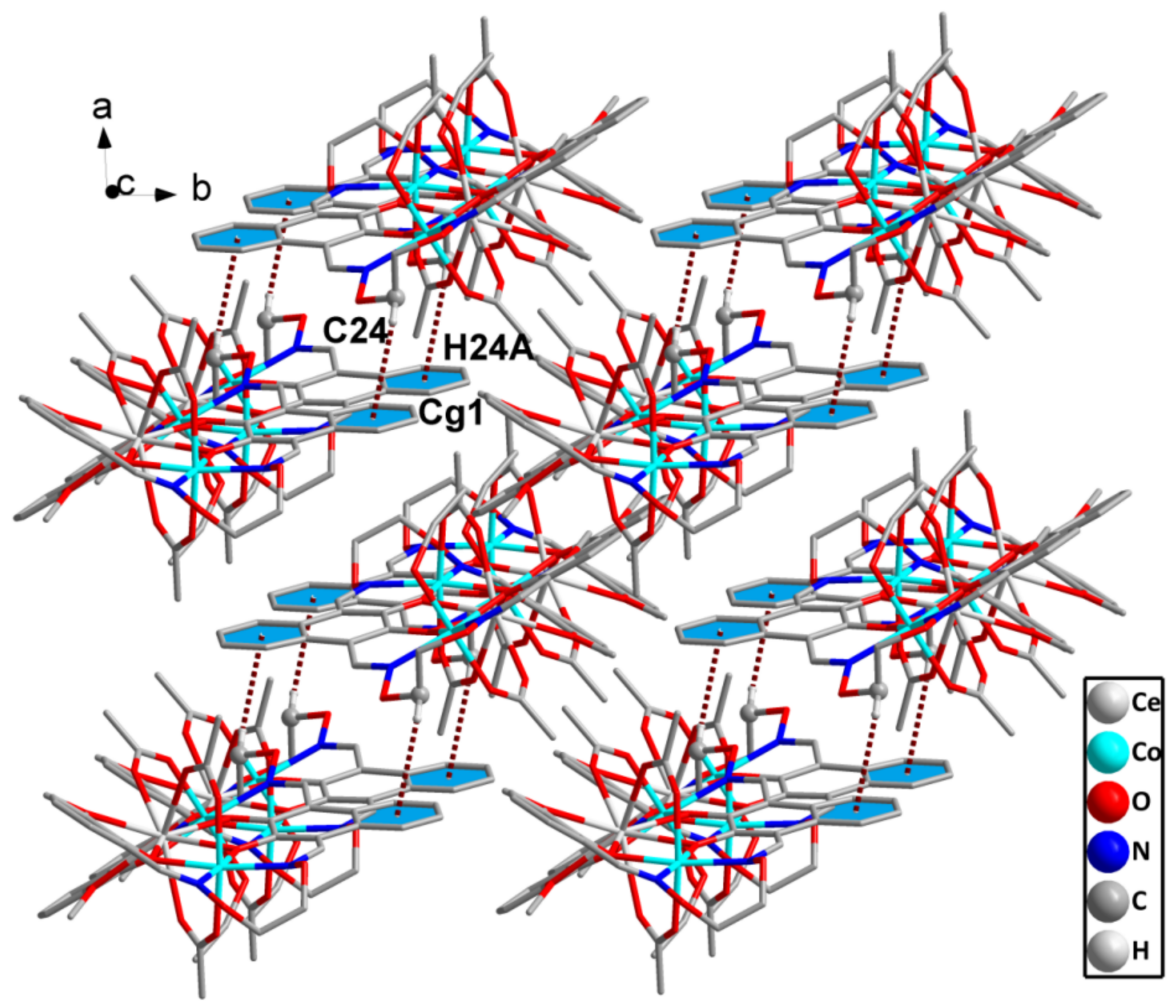
View of 2D supramolecular structure by C–H**···***π* interaction of the Co(II)-Ce(III) complex.

In the crystal structure of the Co(II)-Ce(III) complex, there are four significant intramolecular hydrogen bonds (C9–H9B···O14, C23–H23B···N4, C24–H24B···N3 and C36–H36C···O15) (Figure 5) [65,66,67,68,69]. As illustrated in Figure 6 and Figure 7, three pairs of intermolecular hydrogen bonds (O13–H13···O18, O19–H19A···O16 and C41–H41B···O17) and two intermolecular C-H···Cl interactions (C34–H34A···Cl1 and C36–H36A···Cl1) are formed, respectively [70,71,72,73,74]. Especially, an infinite 2D supramolecular structure are interlinked by one significant C–H···*π* interactions (C24–H24A···Cg1 (C27–C32)) and is shown in Figure 8.

### 2.5. Fluorescence Tests

**Figure 9:**
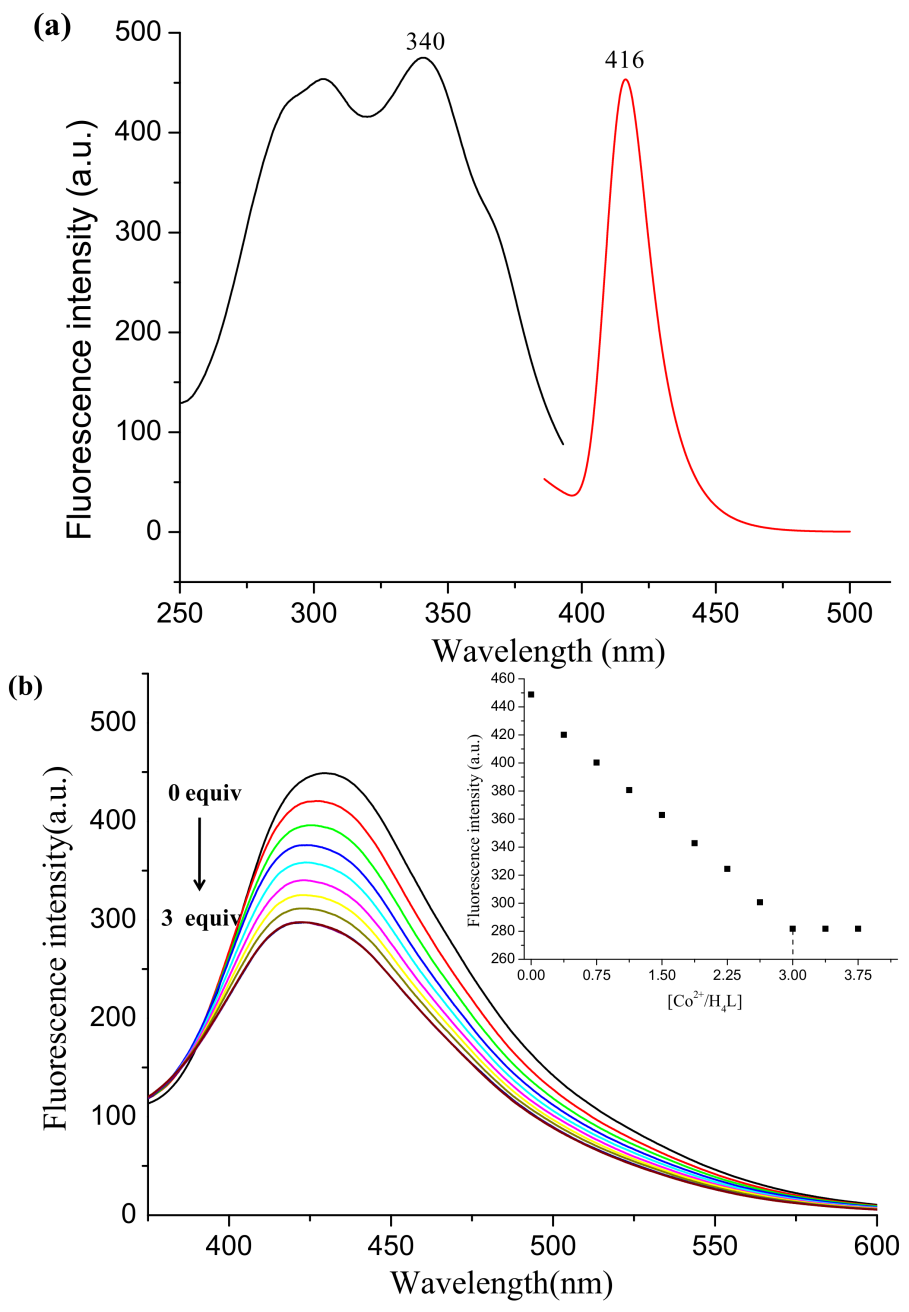
(**a**) The excitation and emission spectra of the Co(II) complex. (**b**) fluorescence emission spectrum changes of H_4_L in methanol solution by the addition of Co(II) ions.

**Figure 10:**
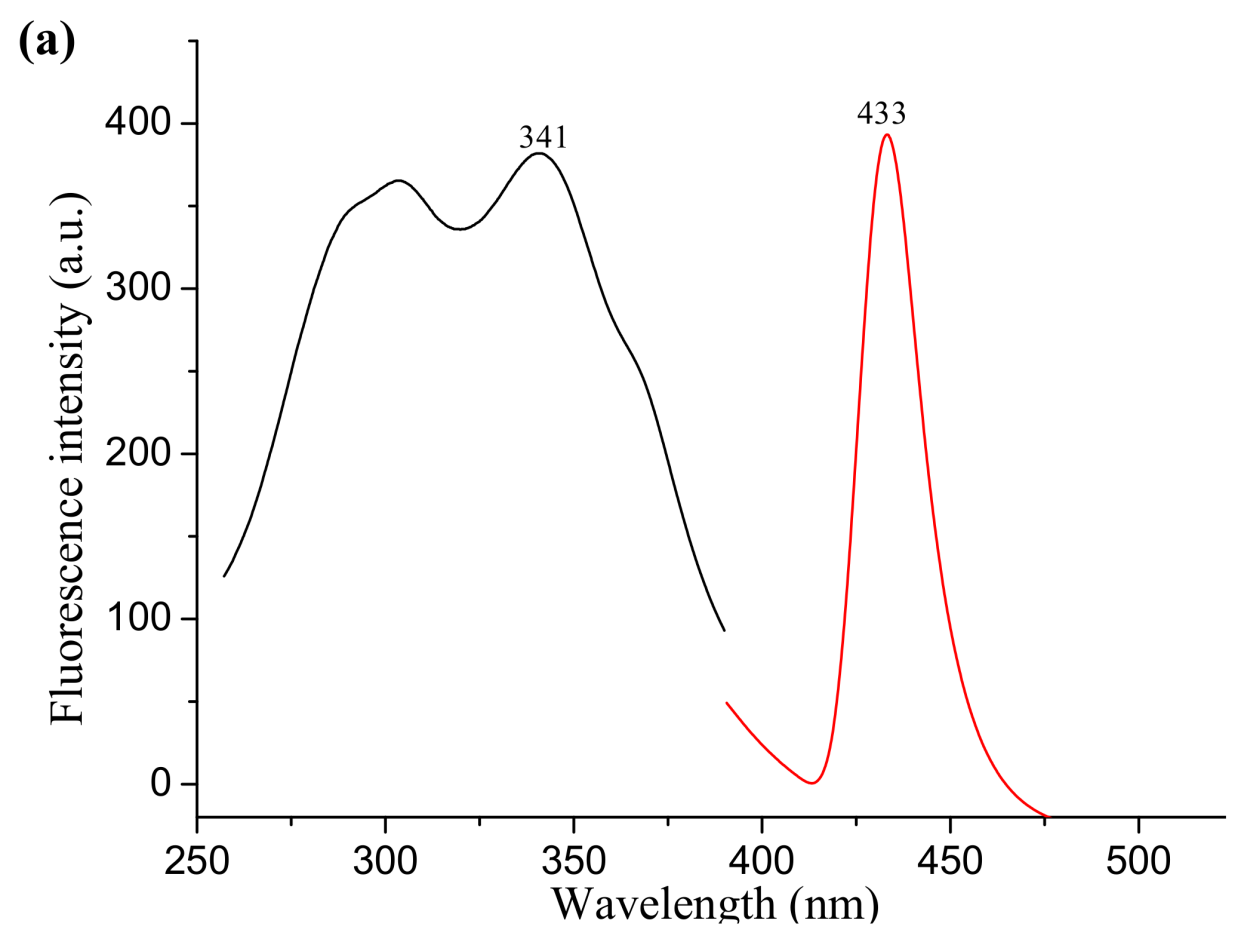

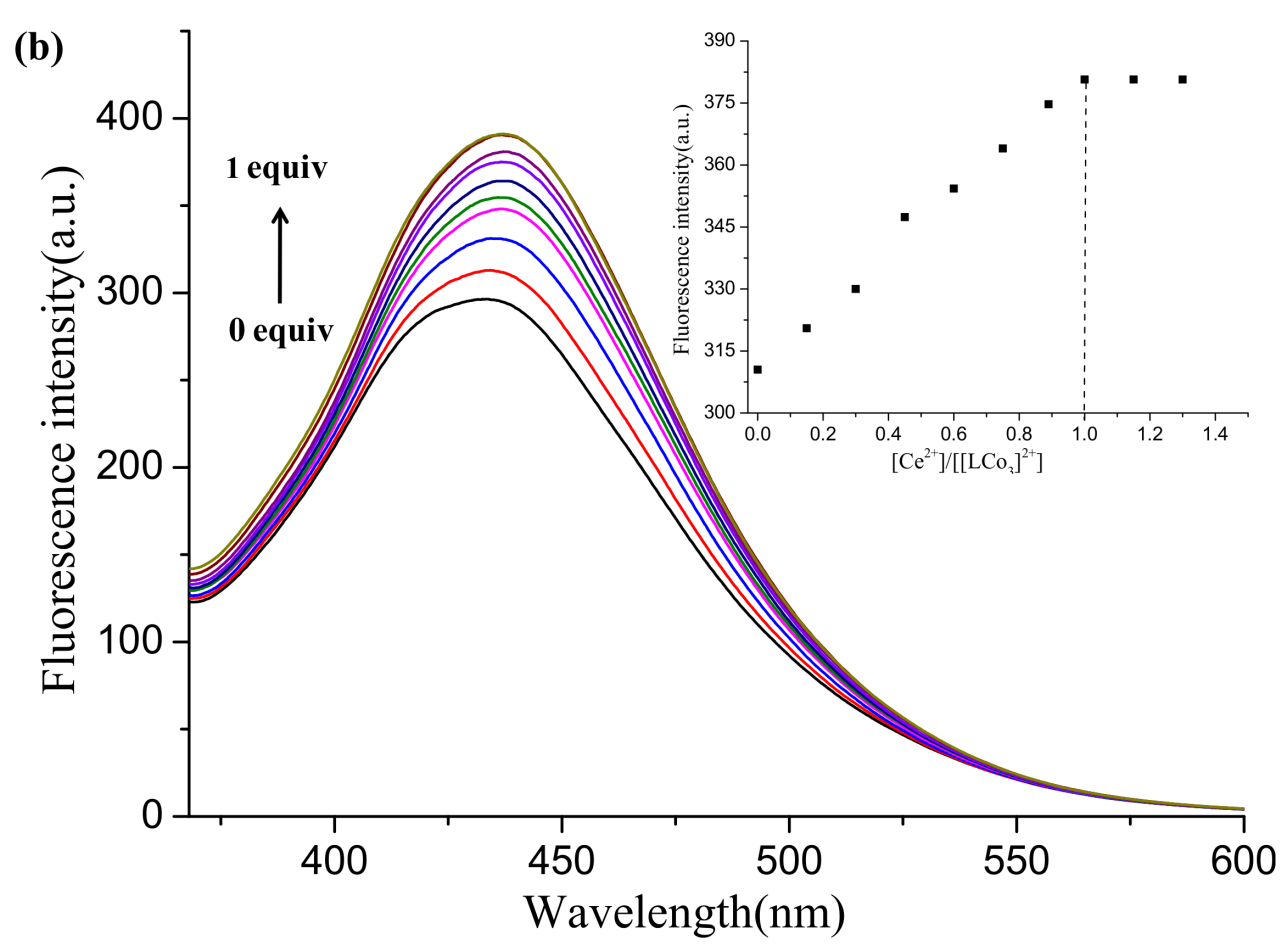
(**a**) The excitation and emission spectra of the Co(II)-Ce(III) complex; (**b**) Fluorescence spectrum changes of Co(II) complex in methanol solution by the addition of Ce(III) ions.

Recently, synthesis of several examples of lanthanide complexes of salamo-type ligands to study their luminescence properties have been reported [75,76]. The fluorescence emission spectra of H_4_L in CHCl_3_:CH_3_OH solution and its corresponding Co(II)-Ce(III) complex in methanol solution were investigated at room temperature. In the fluorescence titration experiment, the emission spectrum of the free ligand H_4_L exhibited broad visible photoluminescence with maximum emission at ca. 416 nm upon excitation at 340 nm (Figure 9a). When Co(II) ions was added, the fluorescence emission intensity quenches. The fluorescence emission intensity of the solution no longer changed after the Co(II) ions was added in excess of three equiv. The spectroscopic titration indicated that the stoichiometric ratio between Co(II) and the ligand H_4_L is 3:1 (Figure 9b). Then, the fluorescence intensity enhances upon addition of Ce(III) ions and showed a maximum at ca. 433 nm upon excitation at 341 nm (Figure 10a). When the amount of Ce(III) ions reaches one equiv, the fluorescence emission intensity of the solution becomes stable. Obviously, the spectroscopic titration indicated that the ratio of the replacement reaction was 1:1 (Figure 10b), which obtained the same conclusion with UV-Vis titration experiments.

## 3. Experimental Section

### 3.1. Materials and Methods

2-Hydroxy-3-methoxybenzaldehyde (99%), methyltrioctylammonium chloride (90%), pyridinium chlorochromate (98%) and boron tribromide (99.9%) were bought from Alfa Aesar (New York, NY, USA) 33 wt % hydrobromic acid solution in acetic acid was purchased from J&K Scientific Ltd. (Beijing, China). The other general reagents and solvents in this work were used directly without further purification in the preparation of the free ligand and its complex. Elemental analyses (C, H and N) were carried out using a VarioEL V3.00 automatic elemental analysis instrument (Elementar, Berlin, Germany).Elemental analyses for metals were performed on an ER/S·WP-1 ICP atomic emission spectrometer (IRIS, Elementar, Berlin, Germany).Melting points were measured using a microscopic melting point apparatus made by Beijing Taike Instrument Limited Company(Beijing, China) and were uncorrected. Infrared spectra were recorded between 500 and 4000 cm^−1^ on a VERTEX 70FT-IR spectrophotometer (Bruker, Billerica, MA, USA)for samples prepared as KBr pellets. UV-Vis spectra in the 250–550 nm range were recorded by a U–3900H spectrometer (Hitachi, Shimadzu, Tokyo, Japan). Fluorescence spectra were taken on a Hitachi F–7000 fluorescence photometer.(Hitachi, Tokyo, Japan). ^1^H-NMR spectra were determined with a German Bruker AVANCE DRX-400 spectrometer (Bruker AVANCE, Billerica, MA, USA). X-ray single crystal structure determination was carried out on a SuperNova Dual (Cu at zero) four-circle diffractometer with graphite monochromated Mo *Kα* radiation (*λ* = 0.71073 Å) at 293.66(10) K (Bruker, Billerica, MA, USA). Reflection data were corrected for Lorentzian and polarization effects and for absorption using the multi-scan method.

### 3.2. Synthesis of the Bi(salamo)-Type Ligand H_4_L

**Scheme 1:**
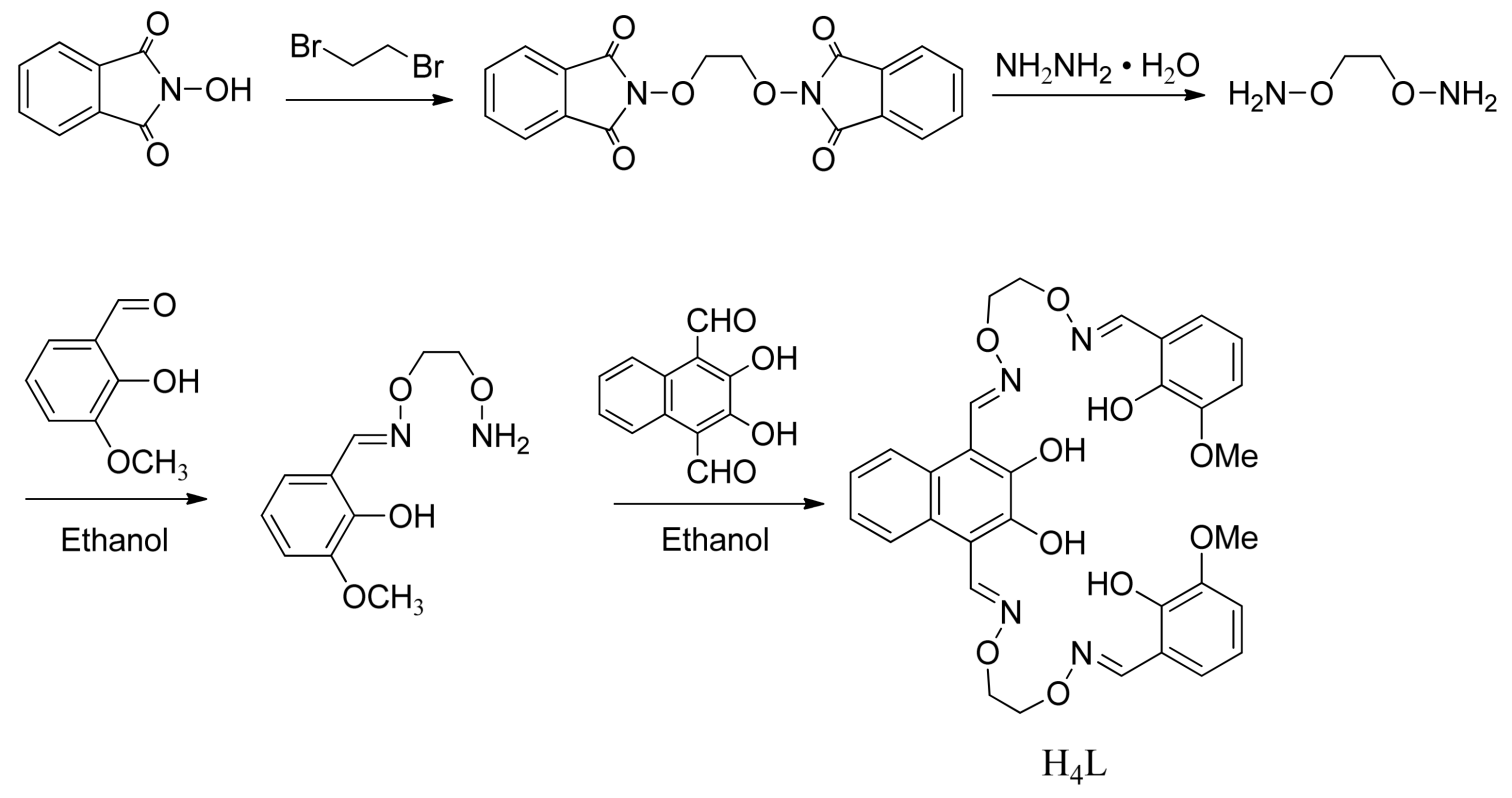
Synthetic route to the bi(salamo)-type ligand H_4_L.

The synthesis of the bi(salamo)-type ligand H_4_L is shown in Scheme 1. 2,3-Dihydroxynaphthalene-1,4-dicarbaldehyde was prepared according to a literature procedure [77]. 2,3-Dihydroxynaphthalene-1,4-dicarbaldehyde and 2-[*O*-(1-ethyloxyamide)]oxime-6-methoxy- phenol were synthesized according to an analogous method [78,79]. A mixed solution of 2,3-dihydroxynaphthalene-1,4-dicarbaldehyde (108.1 mg, 0.50 mmol) in ethanol (10 mL) and 2-[*O*-(1-ethyloxyamide)]oxime-6-methoxyphenol (226.1 mg, 1.00 mmol) in ethanol (10 mL) was heated at 55 °C for 4 h. After cooling to room temperature, the obtained yellow precipitate was filtered off and dried under vacuum to obtain light yellow crystalline solid. Yield: 59%. m.p. 170–171 °C. Anal. Calcd. (%) for C_32_H_32_N_4_O_10_ (632.42): C, 60.75; H, 5.10; N, 8.86. Found (%): C, 60.93; H, 5.23; N, 8.74. ^1^H-NMR (400 MHz, CDCl_3_): no instrument listed in 3.1 *δ* (ppm) = 11.03 (s, 2H), 9.82 (s, 2H), 9.14 (s, 2H), 8.29 (s, 2H), 7.97 (q, *J* = 3.2 Hz, 2H), 7.41 (q, *J* = 6.0, 2.9 Hz, 2H), 7.06–6.68 (m, 6H), 4.58 (t, 8H), 3.89 (s, 6H). UV-Vis [in methanol/chloroform (1:1)], *λ*_max_ (nm) [2.5 × 10^−5^ M]: 342, 360, 375.

### 3.3. Synthesis of the Heterotrinuclear Co(II)-Ce(III) Complex

A mixed solution of Co(OAc)_2_·4H_2_O (4.98 mg, 0.02 mmol) in ethanol (2 mL) and Ce(OAc)_3_·H_2_O (3.35 mg, 0.01 mmol)) in water/methanol (1:1, 2 mL) was added to a solution of the ligand H_4_L (6.32 mg, 0.01 mmol) in dichloromethane (2 mL). Then the resulting mixed solution immediately turned yellow and was stirred at room temperature for 30 min. The mixture was filtered and the filtrate was allowed to stand at room temperature for approximately four weeks, giving colorless prismatic single crystals suitable for X-ray crystallographic analysis. Yield: 37%. Anal. Calcd. (%) for C_42_H_50_ClCo_2_CeN_4_O_18.50_ (1200.29): C, 42.03; H, 4.20; N, 4.67; Co, 9.82; Ce, 11.67. Found (%): C, 42.25; H, 4.47; N, 4.51; Co, 9.64; Ce, 11.49. UV-Vis [in methanol/H_2_O (10:1 *v*/*v*)], *λ*_max_ (nm) [1.0 × 10^−3^ M]: 346, 364, 380.

### 3.4. Crystal Structure Determination and Refinement

The structure was solved by direct methods (SHELX-2014) and refined anisotropically using full-matrix least-squares methods on *F*^2^ with the SHELX-2014 program package. Lp and semi-empirical absorption corrections by SADABS were applied to the intensity data. The non-hydrogen atoms were refined anisotropically except for the solvent molecules of the crystal of the Co(II)-Ce(III) complex. All hydrogen atoms were added in calculated positions. Supplementary crystallographic data for this paper have been deposited at Cambridge Crystallographic Data Centre (1814394) and can be obtained free of charge via www.ccdc.cam.ac.uk/conts/retrieving.html.

## 4. Conclusions

The synthesis, structural characterization, and fluorescence properties of the bis(salamo)-type ligand H_4_L and its corresponding heterotrinuclear Co(II)-Ce(III) complex were described. The X-ray crystal structure revealed that two Co(II) atoms are located in the N_2_O_2_ coordination environment and are both hexacoordinated with slightly distorted octahedral geometries. Simultaneously, the O_6_ cavity of the completely deprotonated (L)^4^^−^ unit is occupied by the Ce(III) atom which is nine-coordinated with a single square antiprismatic geometry. The UV-Vis titration experiments revealed the ratio of the heterotrinuclear Co(II)-Ce(III) complex is 1:2:1 (ligand/Co(II)/Ce(III)). Ethanol as a coordinating solvent participates in the coordination in the Co(II)-Ce(III) complex. The different peak wavelength variation of the heterotrinuclear complex clearly showed the success of transformation from homotrinuclear to heteronuclear complex, which could be used in host-guest systems. Furthermore, the Co(II)-Ce(III) complex showed weak photoluminescence and exhibiting a hypsochromic-shift.

## Figures and Tables

**Table 1:** Selected FT-IR bands for H_4_L and its Co(II)-Ce(III) complex (cm^−1^).

Compound	*ν*(O–H)	*ν*(C=N)	*ν*(Ar–O)
H_4_L	3169	1612	1255
Complex	–	1623	1239

**Table 2:** Crystallographic data and structural refinement paramenters for the Co(II)-Ce(III) complex.

Formula	C_42_H_50_ClCo_2_CeN_4_O_18.50_
Formula weight, g·mol^−1^	1200.29
Temperature, K	293.66(10)
Wavelength, Å	0.71073
Crystal system	Triclinic
Space group	*P*-1
*a*, Å	12.0521(5)
*b*, Å	13.7804(5)
*c*, Å	15.2909(4)
α, °	95.678(3)
β, °	99.471(3)
γ, °	90.210(3)
Volume, Å^3^	2492.13(15)
*Z*	2
Calculated density, mg·m^−3^	1.600
Absorption coefficient, mm^−1^	1.685
*F* (000)	1214
*θ* range for data collection, °	3.390–25.677°
*h*/*k*/*l*(min, max)	−12, 14/−16, 16/−18, 18
Reflections collected	17180
Independent reflections	9641
*R*_int_	0.034
Completeness to *θ* = 26.32	99.78%
Data/restraints/parameters	9641/60/649
Final R indices [I > 2*σ* (I)] ^a^[I > 2*σ*(I)]	*R*_1_ = 0.0519, *wR*_2_ = 0.1377
R indices (all data) ^b^	*R*_1_ = 0.0693, *wR*_2_ = 0.1537
Goodness-of-fit for F^2 c^	1.020
Largest differences peak and hole (e Å^−3^)	1.130 and −1.300

^a^ *R*_1_ = Σ‖*F*_o_| − |*F*_c_‖/Σ|*F*_o_|. b *wR*_2_ = [Σ*w*(*F*_o_^2^ − *F*_c_^2^)^2^*/w*(*F*_o_^2^)^2^]^1/2^, *w* = [*σ*^2^(*F*_o_^2^) + (0.0784*P*)^2^ + 1.3233*P*]^−1^, where *P* = *F*_o_^2^ + 2*F*_c_^2^)*/*3. ^c^ GOF = [Σ*w*(*F*_o_^2^ − *F*_c_^2^)^2^*/*n_obs_ − n_param_)]^1/2^.

**Table 3:** Selected bond lengths (Å) and angles (°) for the Co(II)-Ce(III) complex.

**Bonds Lengths (Å)**			**Bonds Lengths (Å)**		
Co1–O1	2.018(3)	Co1–O2	2.081(4)	Co1–O14	2.088(4)
Co1–O16	2.123(4)	Co1–N1	2.095(5)	Co1–N2	2.116(4)
Co2–O6	2.021(4)	Co2–O7	2.052(4)	Co2–O11	2.058(5)
Co2–O13	2.168(6)	Co2–N3	2.123(5)	Co2–N4	2.139(7)
Ce1–O1	2.421(4)	Ce1–O2	2.461(3)	Ce1–O3	2.688(4)
Ce1–O6	2.467(4)	Ce1–O7	2.523(3)	Ce1–O8	2.655(4)
Ce1–O12	2.437(5)	Ce1–O15	2.528(5)	Ce1–O17	2.526(4)
**Angle (°)**			**Angle (°)**		
O1–Co1–O2	90.51(15)	O1–Co1–O14	87.26(16)	O1–Co1–O16	88.60(16)
O1–Co1–N1	176.65(19)	O1–Co1–N2	83.73(17)	O2–Co1–O14	88.61(15)
O2–Co1–O14	88.61(15)	O2–Co1–O16	85.93(15)	O2–Co1–N1	86.51(17)
O2–Co1–N2	171.58(16)	O14–Co1–O16	173.12(17)	O14–Co1–N1	94.18(18)
O14–Co1–N2	97.21(18)	O16–Co1–N1	89.67(18)	O16–Co1–N2	87.79(18)
N1–Co1–N2	99.1(2)	O1–Ce1–O2	73.22(12)	O1–Ce1–O3	128.52(12)
O1–Ce1–O6	62.41(11)	O1–Ce1–O7	115.41(12)	O1–Ce1–O8	136.97(13)
O1–Ce1–O12	129.53(13)	O1–Ce1–O15	68.24(13)	O1–Ce1–O17	69.66(13)
O2–Ce1–O3	60.44(13)	O2–Ce1–O6	135.35(13)	O2–Ce1–O7	147.78(12)
O2–Ce1–O8	91.55(11)	O2–Ce1–O12	130.03(14)	O2–Ce1–O15	71.51(13)
O2–Ce1–O17	72.66(13)	O3–Ce1–O6	151.91(15)	O3–Ce1–O7	115.69(13)
O3–Ce1–O8	68.54(13)	O3–Ce1–O12	74.57(14)	O3–Ce1–O15	112.71(14)
O3–Ce1–O17	75.97(14)	O6–Ce1–O7	64.67(13)	O6–Ce1–O8	124.04(12)
O6–Ce1–O12	79.85(14)	O6–Ce1–O15	95.34(15)	O6–Ce1–O17	87.10(15)
O7–Ce1–O12	70.43(14)	O7–Ce1–O8	60.38(11)	O7–Ce1–O15	82.89(13)
O7–Ce1–O17	139.42(13)	O8–Ce1–O12	91.27(14)	O8–Ce1–O15	68.78(14)
O8–Ce1–O17	144.42(13)	O12–Ce1–O15	152.40(15)	O12–Ce1–O17	76.59(15)
O15–Ce1–O17	130.63(14)				

**Table 4:** Hydrogen bonds (Å, deg) and C–H···*π* stacking interactions for the Co(II)-Ce(III) complex.

D–H···A	d(D–H)	d(H···A)	d(D···A)	∠DHA	Symmetry Code A
C9–H9B···O14	0.97	2.32	3.249(8)	161	
C36–H36C···O15	0.96	2.20	2.879(16)	126	
C23–H23B···N4	0.97	2.46	2.865(11)	105	
C24–H24B···N3	0.97	2.56	2.957(11)	104	
O13–H13···O18	0.87	1.88	2.733(18)	170	x, y, 1 + z
O19–H19A···O16	0.82	2.05	2.834(9)	160	−x, −y, 1 − z
C34–H34A···Cl1	0.96	2.76	3.446(16)	129	−1 + x, y, z
C36–H36A···Cl1	0.96	2.34	2.955(17)	121	x, y, 1 + z
C41–H41B···O17	0.97	2.38	3.345(16)	173	1 + x, y, z
C24–H24A···Cg1		2.97	3.599(10)	124	−x, −y, −z

Symmetry codes: Cg1 for the Co(II)-Ce(III) complex is the centroid of C27–C32 atoms.

## Supplementary Materials

Supplementary File 1

## References

[1] Chai L.Q., Li Y.X., Chen L.C., Zhang J.Y., Huang J.J. (2016). Synthesis, X-ray structure, spectroscopic, electrochemical properties and DFT calculation of a bridged dinuclear copper(II) complex. Inorg. Chem. Acta.

[2] Ma X., Jiao J.M., Yang J., Huang X.B., Cheng Y.X., Zhu C.J. (2012). Large stokes shift chiral polymers containing (*R*,*R*)-salen-based binuclear boron complex: Synthesis, characterization, and fluorescence properties. Polyhedron.

[3] Zhao L., Wang L., Sun Y.X., Dong W.K., Tang X.L., Gao X.H. (2012). A supramolecular copper(II) complex bearing salen-type bisoxime ligand: Synthesis, structural characterization and thermal property. Synth. React. Inorg. Met-Org. Nano-Met. Chem..

[4] Subramaniam P., Anbarasan S., Devi S.S., Ramdass A. (2016). Modulation of catalytic activity by ligand oxides in the sulfoxidation of phenylmercaptoacetic acids by oxo(salen)chromium(V) complexes. Polyhedron.

[5] Sun Y.X., Zhang S.T., Ren Z.L., Dong X.Y., Wang L. (2013). Synthesis, characterization, and crystal structure of a new supramolecular Cd^II^ complex with halogen-substituted salen-type bisoxime. Synth. React. Inorg. Met.-Org. Nano-Met. Chem..

[6] Shu Y.B., Liu W.S. (2015). Luminescent chiral Eu(III) complexes with enantiopure bis(1H-pyridin-2-one)salen ligands. Polyhedron.

[7] Sun Y.X., Gao X.H. (2011). Synthesis, characterization, and crystal structure of a new Cu^II^ complex with salen-type ligand. Synth. React. Inorg. Met.-Org. Nano-Met. Chem..

[8] Sun Y.X., Xu L., Zhao T.H., Liu S.H., Liu G.H., Dong X.T. (2013). Synthesis and crystal structure of a 3D supramolecular copper(II) complex with 1-(3-{[(E)-3-bromo-5-chloro-2-hydroxybenzylidene]amino}phenyl) ethanone oxime. Synth. React. Inorg. Met.-Org. Nano-Met. Chem.

[9] Song X.Q., Liu P.P., Wang C.Y., Liu Y.A., Liu W.S., Zhang M. (2017). Three sandwich-type zinc(II)–lanthanide(III) clusters: Structures, luminescence and magnetic properties. RSC Adv..

[10] Liu P.P., Sheng L., Song X.Q., Xu W.Y., Liu Y.A. (2015). Synthesis, structure and magnetic properties of a new one dimensional manganese coordination polymer constructed by a new asymmetrical ligand. Inorg. Chim. Acta.

[11] Song X.Q., Liu P.P., Xiao Z.R., Li X., Liu Y.A. (2015). Four polynuclear complexes based on a versatile salicylamide salen-like ligand: Synthesis, structural variations and magnetic properties. Inorg. Chim. Acta.

[12] Liu Y.A., Wang C.Y., Zhang M., Song X.Q. (2017). Structures and magnetic properties of cyclic heterometallic tetranuclear clusters. Polyhedron.

[13] Zhou J.J., Song X.Q., Liu Y.A., Wang X.L. (2017). Substituent-tuned structure and luminescence sensitizing towards Al^3+^ based on phenoxy bridged dinuclear Eu^III^ complexes. RSC Adv..

[14] Dong W.K., Ma J.C., Zhu L.C., Zhang Y. (2016). Nine self-assembled nickel(II)–lanthanide(III) heterometallic complexes constructed from a Salamo-type bisoxime and bearing a N- or O-donor auxiliary ligand: Syntheses, structures and magnetic properties. New J. Chem..

[15] Chin T.K., Endud S., Jamil S., Budagumpi S., Lintang H.O. (2013). Oxidative dimerization of o-aminophenol by heterogeneous mesoporous material modified with biomimetic salen-type copper(II) complex. Catal. Lett..

[16] Li X.Y., Chen L., Gao L., Zhang Y., Akogun S.F., Dong W.K. (2017). Syntheses, crystal structures and catalytic activities of two solvent-induced homotrinuclear Co(II) complexes with a naphthalenediol-based bis(salamo)-type tetraoxime ligand. RSC Adv..

[17] Ömer S., Ümmühan Ö.Ö., Nurgul S., Burcu A., Musa S., Tuncay T., Zeynel S. (2016). A highly selective and sensitive chemosensor derived coumarin-thiazole for colorimetric and fluorimetric detection of CN^−^ ion in DMSO and aqueous solution: Synthesis, sensing ability, Pd(II)/Pt(II) complexes and theoretical studies. Tetrahedron.

[18] Wang L., Li X.Y., Zhao Q., Li L.H., Dong W.K. (2017). Fluorescence properties of heterotrinuclear Zn(II)–M(II) (M = Ca, Sr and Ba) bis(salamo)-type complexes. RSC Adv..

[19] Wu H.L., Bai Y.C., Zhang Y.H., Li Z., Wu M.C., Chen C.Y., Zhang J.W. (2014). Synthesis, crystal structure, antioxidation and DNA-binding properties of a dinuclear copper(II) complex with bis(*N*-salicylidene)-3-oxapentane-1,5-diamine. J. Coord. Chem..

[20] Chen C.Y., Zhang J.W., Zhang Y.H., Yang Z.H., Wu H.L., Pan G.L., Bai Y.C. (2015). Gadolinium(III) and dysprosium(III) complexes with a Schiff base bis(*N*-salicylidene)-3-oxapentane-1,5-diamine: Synthesis, characterization, antioxidation, and DNA-binding studies. J. Coord. Chem..

[21] Wu H.L., Bai Y.H., Zhang Y.H., Pan G.L., Kong J., Shi F.R., Wang X.L. (2014). Two lanthanide(III) complexes based on the Schiff base *N*,*N*′-Bis(salicylidene)-1,5-diamino-3-oxapentane: Synthesis, characterization, DNA-binding properties, and antioxidation. Z. Anorg. Allg. Chem..

[22] Wu H.L., Pan G.L., Wang H., Wang X.L., Bai Y.C., Zhang Y.H. (2014). Study on synthesis, crystal structure, antioxidant and DNA-binding of mono-, di- and poly-nuclear lanthanides complexes with bis(*N*-salicylidene)-3-oxapentane-1,5-diamine. J. Photochem. Photobiol. B Biol..

[23] Wu H.L., Pan G.L., Bai Y.C., Wang H., Kong J., Shi F., Zhang Y.H., Wang X.L. (2014). Preparation, structure, DNA-binding properties, and antioxidant activities of a homodinuclear erbium(III) complex with a pentadentate Schiff base ligand. J. Chem. Res..

[24] Wu H.L., Wang H., Wang X.L., Pan G.L., Shi F.R., Zhang Y.H., Bai Y.C., Kong J. (2014). V-shaped ligand bis(2-benzimidazolylmethyl)amine containing three copper(II) ternary complexes: Synthesis, structure, DNA-binding properties and antioxidant activity. New J. Chem..

[25] Mao S.S., Shen K.S., Shi X.K., Wu H.L., Han X.T., Li C., Huang G.Z. (2018). Synthesis, crystal structure and biological activity of two binuclear Ag(I) complexes with bis-benzimidazole thioether ligands. Inorg. Chim. Acta.

[26] Wu H.L., Pan G.L., Bai Y.C., Wang H., Kong J., Shi F.R., Zhang Y.H., Wang X.L. (2015). Synthesis, structure, antioxidation, and DNA-binding studies of a binuclear ytterbium(III) complex with bis(*N*-salicylidene)-3-oxapentane-1,5-diamine. Res. Chem. Intermed..

[27] Wang F., Xu Y.L., Aderinto S.O., Peng H.P., Zhang H., Wu H.L. (2017). A new highly effective fluorescent probe for Al^3+^ ions and its application in practical samples. J. Photochem. Photobiol. A.

[28] Wu H.L., Bai Y., Yuan J.K., Wang H., Pan G.L., Fan X.Y., Kong J. (2012). A zinc(II) complex with tris(2-(-methyl)benzimidazlylmethyl)amine and salicylate: Synthesis, crystal structure, and DNA-binding. J. Coord. Chem..

[29] Chai L.Q., Wang G., Sun Y.X., Dong W.K., Zhao L., Gao X.H. (2012). Synthesis, crystal structure, and fluorescence of an unexpected dialkoxo-bridged dinuclear copper(II) complex with bis(salen)-type tetraoxime. J. Coord. Chem..

[30] Chai L.Q., Huang J.J., Zhang H.S., Zhang Y.L., Zhang J.Y., Li Y.X. (2014). An unexpected cobalt(III) complex containing a Schiff base ligand: Synthesis, crystal structure, spectroscopic behavior, electrochemical property and SOD-like activity. Spectrochim. Acta Part A.

[31] Chai L.Q., Liu G., Zhang J.Y., Huang J.J., Tong J.F. (2013). Synthesis, crystal structure, fluorescence, electrochemical property, and SOD-like activity of an unexpected nickel(II) complex with a quinazoline-type ligand. J. Coord. Chem..

[32] Chai L.Q., Zhang H.S., Huang J.J., Zhang Y.L. (2015). An unexpected Schiff base-type Ni(II) complex: Synthesis, crystal structures, fluorescence, electrochemical property and SOD-like activities. Spectrochim. Acta Part A.

[33] Chai L.Q., Tang L.J., Chen L.C., Huang J.J. (2017). Structural, spectral, electrochemical and DFT studies of two mononuclear manganese(II) and zinc(II) complexes. Polyhedron.

[34] Chai L.Q., Zhang K.Y., Tang L.J., Zhang J.Y., Zhang H.S. (2017). Two mono- and dinuclear Ni(II) complexes constructed from quinazoline-type ligands: Synthesis, X-ray structures, spectroscopic, electrochemical, thermal, and antimicrobial studies. Polyhedron.

[35] Chen L., Dong W.K., Zhang H., Zhang Y., Sun Y.X. (2017). Structural variation and luminescence properties of tri- and dinuclear Cu^II^ and Zn^II^ complexes constructed from a naphthalenediol-based bis(Salamo)-type ligand. Cryst. Growth Des..

[36] Dong X.Y., Li X.Y., Liu L.Z., Zhang H., Ding Y.J., Dong W.K. (2017). Tri- and hexanuclear heterometallic Ni(II)–M(II) (M = Ca, Sr and Ba) bis(salamo)-type complexes: Synthesis, structure and fluorescence properties. RSC Adv..

[37] Song X.Q., Peng Y.J., Chen G.Q., Wang X.R., Liu P.P., Xu W.Y. (2015). Substituted group-directed assembly of Zn(II) coordination complexes based on two new structural related pyrazolone based Salen ligands: Syntheses, structures and fluorescence properties. Inorg. Chim. Acta.

[38] Wang P., Zhao L. (2016). An infinite 2D supramolecular cobalt(II) complex based on an asymmetric salamo-type ligand: Synthesis, crystal structure, and spectral properties. Synth. React. Inorg. Met.-Org. Nano-Met. Chem..

[39] Dong X.Y., Akogun S.F., Zhou W.M., Dong W.K. (2017). Tetranuclear Zn(II) complex based on an asymmetrical salamo-type chelating ligand: Synthesis, structural characterization, and fluorescence property. J. Chin. Chem. Soc..

[40] Tao C.H., Ma J.C., Zhu L.C., Zhang Y., Dong W.K. (2017). Heterobimetallic 3d–4f Zn(II)–Ln(III) (Ln = Sm, Eu, Tb and Dy) complexes with a N_2_O_4_ bisoxime chelate ligand and a simple auxiliary ligand Py: Syntheses, structures and luminescence properties. Polyhedron.

[41] Dong Y.J., Dong X.Y., Dong W.K., Zhang Y., Zhang L.S. (2017). Three asymmetric salamo-type copper(II) and cobalt(II) complexes: Syntheses, structures, fluorescent properties. Polyhedron.

[42] Dong W.K., Ma J.C., Dong Y.J., Zhao L., Zhu L.C., Sun Y.X., Zhang Y. (2016). Two hetero-trinuclear Zn(II)-M(II) (M = Sr, Ba) complexes based on metallohost of mononuclear Zn(II) complex: Syntheses, structures and fluorescence properties. J. Coord. Chem..

[43] Wu H.L., Wang C.P., Wang F., Peng H.P., Zhang H., Bai Y.C. (2015). A new manganese(III) complex from bis(5-methylsalicylaldehyde)-3-oxapentane-1,5-diamine: Synthesis, characterization, antioxidant activity and luminescence. J. Chin. Chem. Soc..

[44] Wang L., Ma J.C., Dong W.K., Zhu L.C., Zhang Y. (2016). A novel Self–assembled nickel(II)–cerium(III) heterotetranuclear dimer constructed from N_2_O_2_-type bisoxime and terephthalic acid: Synthesis, structure and photophysical properties. Z. Anorg. Allg. Chem..

[45] Dong Y.J., Li X.L., Zhang Y., Dong W.K. (2017). A highly selective visual and fluorescent sensor for Pb^2+^ and Zn^2+^ and crystal structure of Cu^2+^ complex based-on a novel single-armed salamotype bisoxime. Supramol. Chem..

[46] Liu P.P., Wang C.Y., Zhang M., Song X.Q. (2017). Pentanuclear sandwich-type Zn^II^-Ln^III^ clusters based on a new salen-like salicylamide ligand: Structure, near-infrared emission and magnetic properties. Polyhedron.

[47] Li G., Hao J., Liu L.Z., Zhou W.M., Dong W.K. (2017). Syntheses, crystal structures and thermal behaviors of two supramolecular salamo-type cobalt(II) and zinc(II) complexes. Crystals.

[48] Wang B.J., Dong W.K., Zhang Y., Akogun S.F. (2017). A novel relay-sensor for highly sensitive and selective detection of Zn^2+^/Pic^−^ and fluorescence on/off switch response of H^+^/OH^−^. Sens. Actuators B Chem..

[49] Dong W.K., Li G., Wang Z.K., Dong X.Y. (2014). A novel trinuclear cobalt(II) complex derived from an asymmetric salamo-type N_2_O_3_ bisoxime chelate ligand: Synthesis, structure and optical properties. Spectrochim. Acta Part A.

[50] Dong X.Y., Gao L., Wang F., Zhang Y., Dong W.K. (2017). Tri- and mono-nuclear zinc(II) complexes based on half- and mono-salamo chelating ligands. Crystals.

[51] Yu B., Li C.Y., Sun Y.X., Jia H.R., Guo J.Q., Li J. (2017). A new azine derivative colorimetric and fluorescent dual-channel probe for cyanide detection. Spectrochim. Acta Part A.

[52] Dong W.K., Lan P.F., Zhou W.M., Zhang Y. (2016). Salamo-type trinuclear and tetranuclear cobalt (II) complexes based on a new asymmetry salamo-type ligand: Syntheses, crystal structures, and fluorescence. J. Coord. Chem..

[53] Xu L., Zhu L.C., Ma J.C., Zhang Y., Zhang J., Dong W.K. (2015). Syntheses, structures and spectral properties of mononuclear Cu^II^ and dimeric Zn^II^ complexes based on an asymmetric Salamo-type N_2_O_2_ ligand. Z. Anorg. Allg. Chem..

[54] Wang F., Gao L., Zhao Q., Zhang Y., Dong W.K., Ding Y.J. (2018). A highly selective fluorescent chemosensor for CN^−^ based on a novel bis(salamo)-type tetraoxime ligand. Spectrochim. Acta Part A.

[55] Gao L., Wang F., Zhao Q., Zhang Y., Dong W.K. (2018). Mononuclear Zn(II) and trinuclear Ni(II) complexes derived from a coumarin-containing N_2_O_2_ ligand: Syntheses, crystal structures and fluorescence properties. Polyhedron.

[56] Dong W.K., Du W., Zhang X.Y., Li G., Dong X.Y. (2014). Synthesis, crystal structure and spectral properties of a supramolecular trinuclear nickel(II) complex with 5-methoxy-4′-bromo-2,2′-[ethylenedioxybis(nitrilomethylidyne)]diphenol. Spectrochim. Acta Part A.

[57] Dong W.K., Zheng S.S., Zhang J.T., Zhang Y., Sun Y.X. (2017). Luminescent properties of heterotrinuclear 3d–4f complexes constructed from a naphthalenediol-based acyclic bis(salamo)-type ligand. Spectrochim. Acta Part A.

[58] Dong Y.J., Ma J.C., Zhu L.C., Dong W.K., Zhang Y. (2017). Four 3d–4f heteromultinuclear zinc(II)–lanthanide(III) complexes constructed from a distinct hexadentate N_2_O_2_-type ligand: Syntheses, structures and luminescence properties. J. Coord. Chem..

[59] Ma J.C., Dong X.Y., Dong W.K., Zhang Y., Zhu L.C., Zhang J.T. (2016). An unexpected dinuclear Cu(II) complex with a bis(Salamo) chelating ligand: Synthesis, crystal structure, and photophysical properties. J. Coord. Chem..

[60] Dong W.K., Ma J.C., Dong Y.J., Zhu L.C., Zhang Y. (2016). Di- and tetranuclear heterometallic 3d–4f cobalt(II)–lanthanide(III) complexes derived from a hexadentate bisoxime: Syntheses, structures and magnetic properties. Polyhedron.

[61] Dong W.K., Ma J.C., Zhu L.C., Zhang Y. (2016). Self-assembled zinc(II)-lanthanide(III) heteromultinuclear complexes constructed from 3-MeOsalamo ligand: Syntheses, structures and luminescent properties. Cryst. Growth Des..

[62] Zhang H., Dong W.K., Zhang Y., Akogun S.F. (2017). Naphthalenediol-based bis(Salamo)-type homo- and heterotrinuclear cobalt(II) complexes: Syntheses, structures and magnetic properties. Polyhedron.

[63] Li L.H., Dong W.K., Zhang Y., Akogun S.F., Xu L. (2017). Syntheses, structures and catecholase activities of homo- and hetero-trinuclear cobalt(II) complexes constructed from an acyclic naphthalenediolbased bis(Salamo)-type ligand. Appl. Organomet. Chem..

[64] Wang L., Hao J., Zhai L.X., Zhang Y., Dong W.K. (2017). Synthesis, crystal structure, luminescence, electrochemical and antimicrobial properties of bis(salamo)-based Co(II) complex. Crystals.

[65] Li X.Y., Kang Q.P., Liu L.Z., Ma J.C., Dong W.K. (2018). Trinuclear Co(II) and mononuclear Ni(II) Salamo-type bisoxime coordination compounds. Crystals.

[66] Peng Y.D., Li X.Y., Kang Q.P., An G.X., Zhang Y., Dong W.K. (2018). Synthesis and fluorescence properties of asymmetrical Salamo-type tetranuclear zinc(II) complex. Crystals.

[67] Gao L., Liu C., Wang F., Dong W.K. (2018). Tetra-, penta- and hexa-coordinated transition metal complexes constructed from coumarin-containing N_2_O_2_ ligand. Crystals.

[68] Sun Y.X., Li C.Y., Yang C.J., Zhao Y.Y., Guo J.Q., Yu B. (2016). Two Cu(II) complexes with Schiff base ligands: Syntheses, crystal structures, spectroscopic properties and substituent effect. Chin. J. Inorg. Chem..

[69] Yu B., Sun Y.X., Yang C.J., Guo J.Q., Li J. (2017). Synthesis and crystal structures of an unexpected tetranuclear zinc(II) complex and a benzoquinone compound derived from Zn^II^- and Cd^II^-promoted reactivity of Schiff base ligands. Z. Anorg. Allg. Chem..

[70] Sun Y.X., Lu R.E., Li X.R., Zhao Y.Y., Li C.Y. (2015). A Schiff base ligand containing oxime group and its Cu(II) complex: Syntheses and supramolecular structures. Chin. J. Inorg. Chem..

[71] Guo J.Q., Sun Y.X., Yu B., Li J., Jia H.R. (2017). Syntheses, crystal structures and spectroscopic properties of copper(II) and nickel(II) complexes with oxime-type Schiff base ligands. Chin. J. Inorg. Chem..

[72] Xu Y.L., Mao S.S., Shen K.S., Shi X.K., Wu H.L., Tang X. (2018). Different structures of two Cu(I) complexes constructed by bridging 2,2-(1,4-butanediyl)bis-1,3-benzoxazole ligand: Syntheses, structures and properties. Inorg. Chim. Acta.

[73] Sun Y.X., Zhao Y.Y., Li C.Y., Yu B., Guo J.Q., Li J. (2016). Supramolecular cobalt(II) and copper(II) complexes with Schiff base ligand: Syntheses, characterization and crystal structures. Chin. J. Inorg. Chem..

[74] Jia H.R., Li J., Sun Y.X., Guo J.Q., Yu B., Wen N., Xu L. (2017). Two supramolecular cobalt(II) complexes: Syntheses, crystal structures, spectroscopic behaviors, and counter anion effects. Crystals.

[75] Zheng S.S., Dong W.K., Zhang Y., Chen L., Dong Y.G. (2017). Four salamo-type 3d–4f hetero-bimetallic [Zn^II^Ln^III^] complexes: Syntheses, crystal structures, and luminescent and magnetic properties. New J. Chem..

[76] Hao J., Li L.H., Zhang J.T., Akogun S.F., Wang L., Dong W.K. (2017). Four homo- and hetero-bismetallic 3d/3d-2s complexes constructed from a naphthalenediol-based acyclic bis(salamo)-type tetraoxime ligand. Polyhedron.

[77] Akine S., Sairenji S., Taniguchi T., Nabeshima T. (2013). Stepwise helicity inversions by multisequential metal exchange. J. Am. Chem. Soc..

[78] Hao J., Liu L.Z., Dong W.K., Zhang J., Zhang Y. (2017). Three multinuclear Co(II), Zn(II) and Cd(II) complexes based on a single-armed salamo-type bisoxime: Syntheses, structural characterizations and fluorescent properties. J. Coord. Chem..

[79] Hao J., Li X.Y., Zhang Y., Dong W.K. (2018). A reversible bis(Salamo)-based fluorescence sensor for selective detection of Cd^2+^ in water-containing systems and food samples. Materials.

