# Supplementary material for: Synthesis and Fluorescence Properties of a New Heterotrinuclear Co(II)-Ce(III)Complex Constructed from a bis(salamo)-Type Tetraoxime Ligand
Source: Molecules. 2018 Mar 31;23(4):804. doi: 10.3390/molecules23040804 (PMC6017765; doi:10.3390/molecules23040804)

checkCIF/PLATON report

You have not supplied any structure factors. As a result the full set of tests cannot be run.

THIS REPORT IS FOR GUIDANCE ONLY. IF USED AS PART OF A REVIEW PROCEDURE FOR PUBLICATION, IT SHOULD NOT REPLACE THE EXPERTISE OF AN EXPERIENCED CRYSTALLOGRAPHIC REFEREE.

No syntax errors found. CIF dictionary Interpreting this report

Datablock: d903

```
Bond precision:  C-C = 0.0098 A          Wavelength=0.71073

Cell:          a=12.0521(5)      b=13.7804(5)     c=15.2909(4)
               alpha=95.678(3)   beta=99.471(3)   gamma=90.210(3)
Temperature:   294 K
```

	Calculated	Reported
Volume	2492.13(15)	2492.13(15)
Space group	P -1	P -1
Hall group	-P 1	-P 1
Moiety formula	2(C40 H43 Ce Co2 N4 O17), C H2 Cl2, 3(C H4 O)	C40 H43 Ce Co2 N4 O17, 0.5(C H2 Cl2), 1.5(C H4 O)
Sum formula	C84 H100 Ce2 Cl2 Co4 N8 O37	C42 H50 Ce Cl Co2 N4 O18.50
Mr	2400.59	1200.29
Dx,g cm-3	1.600	1.600
Z	1	2
Mu (mm-1)	1.685	1.685
F000	1214.0	1214.0
F000’	1215.74	
h,k,lmax	14,16,18	14,16,18
Nref	9481	9461
Tmin,Tmax		0.879,1.000
Tmin’		

```
Correction method= # Reported T Limits: Tmin=0.879 Tmax=1.000
AbsCorr = MULTI-SCAN

Data completeness= 0.998           Theta(max)= 25.677

R(reflections)= 0.0519( 7494)     wR2(reflections)= 0.1537( 9461)

S = 1.025                  Npar= 649
```

The following ALERTS were generated. Each ALERT has the format
test-name_ALERT_alert-type_alert-level.
Click on the hyperlinks for more details of the test.

Alert level C

```
PLAT053_ALERT_1_C Minimum Crystal Dimension Missing (or Error) ...     Please Check
PLAT054_ALERT_1_C Medium  Crystal Dimension Missing (or Error) ...     Please Check
PLAT055_ALERT_1_C Maximum Crystal Dimension Missing (or Error) ...     Please Check
PLAT220_ALERT_2_C Non-Solvent  Resd 1  C   Ueq(max)/Ueq(min) Range        5.6 Ratio
PLAT222_ALERT_3_C Non-Solv.  Resd 1  H   Uiso(max)/Uiso(min) Range        5.6 Ratio
PLAT230_ALERT_2_C Hirshfeld Test Diff for   C1        --C2        .       6.3 s.u.
PLAT234_ALERT_4_C Large Hirshfeld Difference O9        --C23             0.16 Ang.
PLAT241_ALERT_2_C High   'MainMol' Ueq as Compared to Neighbors of        C25 Check
PLAT242_ALERT_2_C Low    'MainMol' Ueq as Compared to Neighbors of        Co2 Check
PLAT242_ALERT_2_C Low    'MainMol' Ueq as Compared to Neighbors of        C33 Check
PLAT342_ALERT_3_C Low Bond Precision on  C-C Bonds ...............    0.00985 Ang.
PLAT412_ALERT_2_C Short Intra XH3 .. XHn   H35A      ..H36B      ..      1.88 Ang.
PLAT412_ALERT_2_C Short Intra XH3 .. XHn   H36B      ..H38B      ..      1.84 Ang.
PLAT414_ALERT_2_C Short Intra D-H..H-X     H13       ..H35B      ..      1.96 Ang.
```

Alert level G

```
PLAT002_ALERT_2_G Number of Distance or Angle Restraints on AtSite          7 Note
PLAT003_ALERT_2_G Number of Uiso or Uij Restrained non-H Atoms ...         10 Report
PLAT007_ALERT_5_G Number of Unrefined Donor-H Atoms ..............          3 Report
PLAT012_ALERT_1_G No       _shelx_res_checksum Found in CIF ......     Please Check
PLAT042_ALERT_1_G Calc. and Reported MoietyFormula Strings  Differ     Please Check
PLAT045_ALERT_1_G Calculated and Reported Z Differ by a Factor ...       0.50 Check
PLAT154_ALERT_1_G The s.u.'s on the Cell Angles are Equal ..(Note)      0.003 Degree
PLAT172_ALERT_4_G The CIF-Embedded .res File Contains DFIX Records          6 Report
PLAT186_ALERT_4_G The CIF-Embedded .res File Contains ISOR Records          3 Report
PLAT187_ALERT_4_G The CIF-Embedded .res File Contains RIGU Records          2 Report
PLAT300_ALERT_4_G Atom Site Occupancy of Cl1        Constrained at        0.5 Check
PLAT300_ALERT_4_G Atom Site Occupancy of Cl2        Constrained at        0.5 Check
PLAT300_ALERT_4_G Atom Site Occupancy of C41        Constrained at        0.5 Check
PLAT300_ALERT_4_G Atom Site Occupancy of H41A       Constrained at        0.5 Check
PLAT300_ALERT_4_G Atom Site Occupancy of H41B       Constrained at        0.5 Check
PLAT300_ALERT_4_G Atom Site Occupancy of O18        Constrained at        0.5 Check
PLAT300_ALERT_4_G Atom Site Occupancy of C42        Constrained at        0.5 Check
PLAT300_ALERT_4_G Atom Site Occupancy of H18A       Constrained at        0.5 Check
PLAT300_ALERT_4_G Atom Site Occupancy of H42A       Constrained at        0.5 Check
PLAT300_ALERT_4_G Atom Site Occupancy of H42B       Constrained at        0.5 Check
PLAT300_ALERT_4_G Atom Site Occupancy of H42C       Constrained at        0.5 Check
PLAT302_ALERT_4_G Anion/Solvent/Minor-Residue Disorder (Resd  2  )       100% Note
PLAT302_ALERT_4_G Anion/Solvent/Minor-Residue Disorder (Resd  3  )       100% Note
PLAT304_ALERT_4_G Non-Integer Number of Atoms in ...... Resd  2          2.50 Check
PLAT333_ALERT_2_G Large Aver C6-Ring C-C Dist. C12      -C28             1.42 Ang.
PLAT343_ALERT_2_G Unusual sp3      Angle Range in Main Residue for        C35 Check
PLAT395_ALERT_2_G Deviating  X-O-Y    Angle From 120 for O4             111.2 Degree
PLAT395_ALERT_2_G Deviating  X-O-Y    Angle From 120 for O5             110.9 Degree
PLAT395_ALERT_2_G Deviating  X-O-Y    Angle From 120 for O9             111.2 Degree
PLAT395_ALERT_2_G Deviating  X-O-Y    Angle From 120 for O10            109.5 Degree
PLAT432_ALERT_2_G Short Inter X...Y Contact  Cl1      ..C36              2.95 Ang.
PLAT790_ALERT_4_G Centre of Gravity not Within Unit Cell: Resd.  #          4 Note
              C H4 O
PLAT860_ALERT_3_G Number of Least-Squares Restraints .............         60 Note
```

---

0 **ALERT level A** = Most likely a serious problem - resolve or explain
0 **ALERT level B** = A potentially serious problem, consider carefully

```
14 ALERT level C = Check. Ensure it is not caused by an omission or oversight
33 ALERT level G = General information/check it is not something unexpected

 7 ALERT type 1 CIF construction/syntax error, inconsistent or missing data
17 ALERT type 2 Indicator that the structure model may be wrong or deficient
 3 ALERT type 3 Indicator that the structure quality may be low
19 ALERT type 4 Improvement, methodology, query or suggestion
 1 ALERT type 5 Informative message, check
```

It is advisable to attempt to resolve as many as possible of the alerts in all categories. Often the minor alerts point to easily fixed oversights, errors and omissions in your CIF or refinement strategy, so attention to these fine details can be worthwhile. In order to resolve some of the more serious problems it may be necessary to carry out additional measurements or structure refinements. However, the purpose of your study may justify the reported deviations and the more serious of these should normally be commented upon in the discussion or experimental section of a paper or in the "special_details" fields of the CIF. checkCIF was carefully designed to identify outliers and unusual parameters, but every test has its limitations and alerts that are not important in a particular case may appear. Conversely, the absence of alerts does not guarantee there are no aspects of the results needing attention. It is up to the individual to critically assess their own results and, if necessary, seek expert advice.

Publication of your CIF in IUCr journals

A basic structural check has been run on your CIF. These basic checks will be run on all CIFs submitted for publication in IUCr journals (*Acta Crystallographica*, *Journal of Applied Crystallography*, *Journal of Synchrotron Radiation*); however, if you intend to submit to *Acta Crystallographica Section C* or *E* or *IUCrData*, you should make sure that full publication checks are run on the final version of your CIF prior to submission.

Publication of your CIF in other journals

Please refer to the *Notes for Authors* of the relevant journal for any special instructions relating to CIF submission.

PLATON version of 13/12/2017; check.def file version of 12/12/2017

Datablock d903 - ellipsoid plot

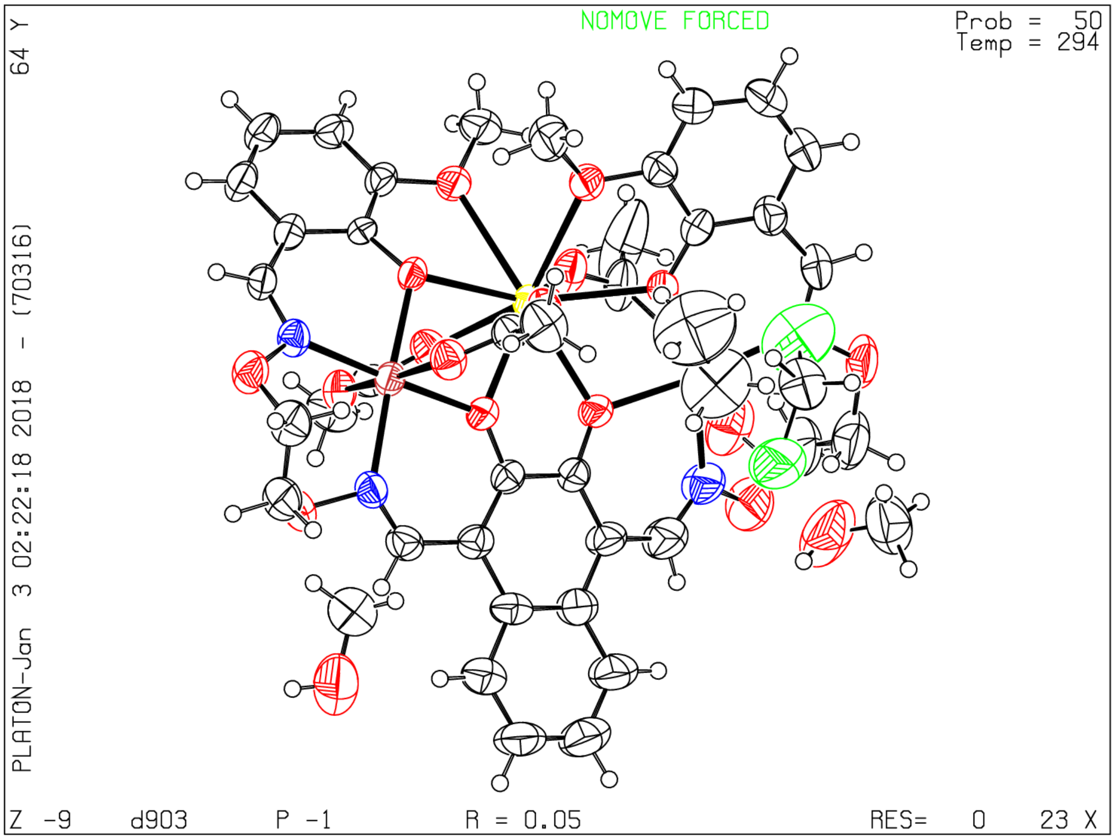

Supplement: Supplementary File 1 [file molecules-23-00804-s001.zip › CheckCif/Complex checkcif report.pdf]
